# Homeostatic cytokines reciprocally modulate the emergence of prenatal effector PLZF^+^CD4^+^ T cells in humans

**DOI:** 10.1172/jci.insight.164672

**Published:** 2023-11-22

**Authors:** Veronica Locher, Sara Park, Daniel G. Bunis, Stephanie Makredes, Margareta Mayer, Trevor D. Burt, Gabriela K. Fragiadakis, Joanna Halkias

**Affiliations:** 1 Division of Neonatology, Department of Pediatrics, and; 2 Eli and Edythe Broad Center of Regeneration Medicine and Stem Cell Research, UCSF, San Francisco, California, USA.; 3 Committee on Immunology, University of Chicago, Chicago, Illinois, USA.; 4 Bakar ImmunoX Initiative and; 5 CoLabs, UCSF, San Francisco, California, USA.; 6 Division of Neonatology and the Children’s Health & Discovery Initiative, Department of Pediatrics, Duke University School of Medicine, Durham, North Carolina, USA.; 7 Division of Rheumatology, Department of Medicine, UCSF, San Francisco, California, USA.

**Keywords:** Development, Immunology, Cytokines, T cell development

## Abstract

The development of human prenatal adaptive immunity progresses faster than previously appreciated, with the emergence of memory CD4^+^ T cells alongside regulatory T cells by midgestation. We previously identified a prenatal specific population of promyelocytic leukemia zinc finger–positive (PLZF^+^) CD4^+^ T cells with heightened effector potential that were enriched in the developing intestine and accumulated in the cord blood of infants exposed to prenatal inflammation. However, the signals that drive their tissue distribution and effector maturation are unknown. Here, we define the transcriptional and functional heterogeneity of human prenatal PLZF^+^CD4^+^ T cells and identify the compartmentalization of T helper–like (Th-like) effector function across the small intestine (SI) and mesenteric lymph nodes (MLNs). IL-7 was more abundant in the SI relative to the MLNs and drove the preferential expansion of naive PLZF^+^CD4^+^ T cells via enhanced STAT5 and MEK/ERK signaling. Exposure to IL-7 was sufficient to induce the acquisition of CD45RO expression and rapid effector function in a subset of PLZF^+^CD4^+^ T cells, identifying a human analog of memory phenotype CD4^+^ T cells. Further, IL-7 modulated the differentiation of Th1- and Th17-like PLZF^+^CD4^+^ T cells and thus likely contributes to the anatomic compartmentalization of human prenatal CD4^+^ T cell effector function.

## Introduction

Pregnancy is a critical period of human immune development. Unlike mice, humans are born with a complete immune cell compartment, and T cells are present in lymphoid and nonlymphoid organs by 12–14 weeks of gestation. Specifically, αβ and γδ T cells emerge simultaneously during prenatal thymopoiesis and, along with regulatory T (Treg) cells, are evident in mucosal tissues by the second trimester of pregnancy (1–4). Thus, in humans, the critical early-life window of development, in which the immune system is highly receptive to environmental instruction (5), begins in utero.

Growing evidence indicates that T cells in early life are a specialized population adapted to the unique demands of the perinatal period (6). These include the need for tolerance to self-antigens and maternal antigens in utero and a simultaneous requirement for the generation of a protective immune compartment to face the multitude of environmental antigens encountered after birth. The survival of a healthy newborn requires the concurrent generation of both regulatory and protective T cells, adding a layer of complexity to the development of human prenatal adaptive immunity.

The priming and differentiation of naive T cells upon encounter with cognate antigen in the context of MHC, costimulatory signals, and specific cytokine milieux result in the generation of distinct T helper (Th) cell subsets with characteristic transcriptional and cytokine production profiles (7). In addition to foreign antigen–specific memory Th cells, murine studies have identified memory phenotype CD4^+^ T cells, which arise in response to interactions with self-peptide MHC in combination with homeostatic cytokines in the setting of lymphopenia-induced proliferation (8, 9). Early-life CD8^+^ T cells have a cell-intrinsic propensity to become virtual memory cells and constitute the early effector response to infection in adulthood (10). Thus, the human in utero environment with limited yet impending exposure to a multitude of foreign antigens after birth, and a peripheral compartment largely void of immune cells, is ideally positioned for the generation of memory phenotype cells with innate-like effector functions to provide rapid protection at mucosal surfaces. However, whether these cells are present in humans is not known.

The majority of T cells in mice and humans reside in lymphoid and mucosal tissues, with a heterogeneous distribution of subsets and functions across specific sites (11, 12). In the mature immune system, the anatomic location of T cells is critical to their differentiation and function. It is likely that the developing prenatal immune system similarly integrates local cues to induce the differentiation of cell types tailored to meet the needs of the tissue they reside in. Naive T cells predominate in most infant and pediatric tissues, yet memory phenotype CD4^+^ T cells with rapid effector capability are evident in the small intestine (SI) by the second trimester of pregnancy (13–16). We recently demonstrated that the majority of these intestinal memory CD4^+^ T cells express the transcription factor promyelocytic leukemia zinc finger (PLZF), and though present in lymphoid and nonlymphoid tissues, prenatal PLZF^+^CD4^+^ T cells specifically accumulated in the intestine and were superior producers of Th1 cytokines as compared with PLZF^–^CD4^+^ T cells (14). Moreover, PLZF^+^CD4^+^ T cells were enriched in the cord blood of infants exposed to prenatal inflammatory pathologies, underscoring the clinical relevance of understanding the signals that drive the maturation and compartmentalization of these human effector T cells in utero.

To better understand how the prenatal environment shapes the developing human CD4^+^ T cell compartment, we set out to identify the cues that drive T cell maturation and function during normal development. We provide evidence of the functional heterogeneity and spatial segregation of memory PLZF^+^CD4^+^ T cells between the lamina propria of the SI and the intestinal draining mesenteric lymph nodes (MLNs), revealing a diversity of Th-like effector function that parallels that of conventional CD4^+^ T cells. We further define tissue-specific differences in homeostatic cytokines and identify IL-7 as a critical driver in the distribution and functional maturation of prenatal effector PLZF^+^CD4^+^ T cells, including the generation of memory phenotype CD4^+^ T cells in response to lymphopenia-induced proliferation.

## Results

### Prenatal PLZF^+^CD4^+^ T cells are functionally heterogeneous and display spatially compartmentalized effector function.

**Figure 1:**
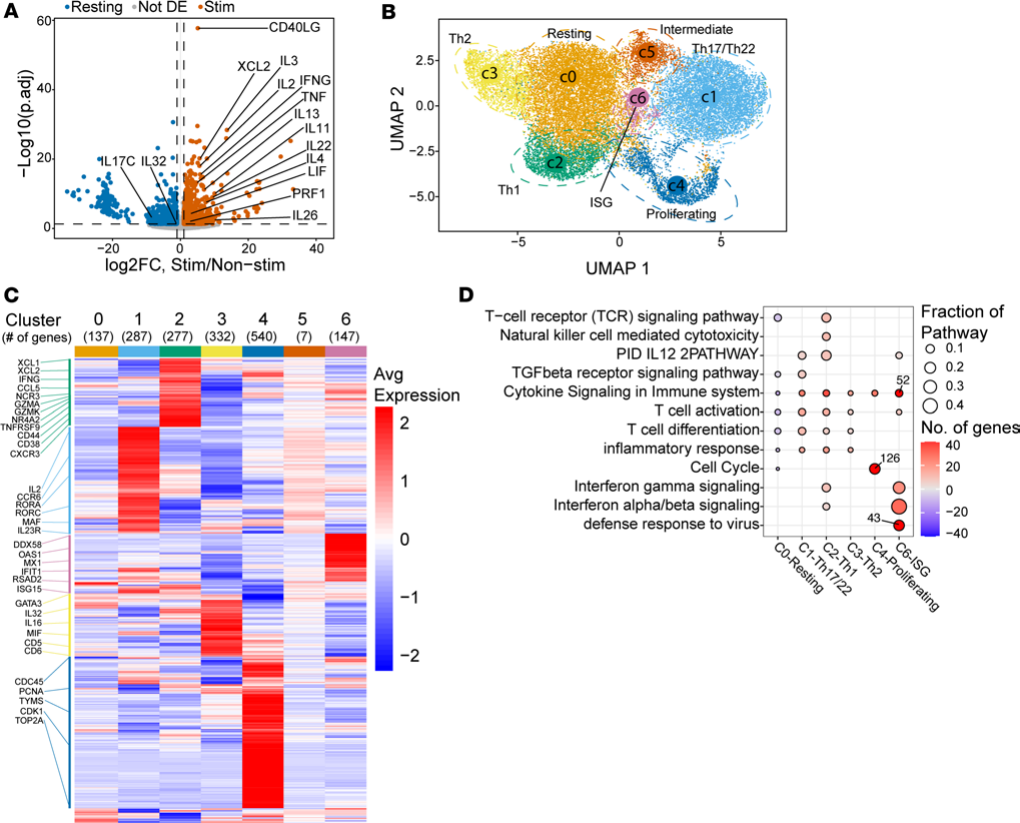
Prenatal PLZF^+^CD4^+^ T cells are a heterogeneous population. (**A**) Population-wide RNA-Seq of prenatal small intestine (SI) PLZF^+^CD4^+^ T cells (*n* = 5) identifies transcriptional expression of numerous cytokines after PMA/ionomycin stimulation. Volcano plot of enriched (orange) and depleted (blue) differentially expressed (DE) gene transcripts (log_2_ fold-change > 1, FDR ≤ 0.05) within stimulated SI PLZF^+^CD4^+^ T cells. (**B**) Single-cell transcriptional analysis of memory Vα7.2^–^PLZF^+^CD4^+^TCRαβ^+^ T cells from the prenatal SI of 3 donors identifies 7 distinct clusters. Uniform manifold approximation and projection (UMAP) visualization of identified clusters within SI PLZF^+^CD4^+^ T cells in which each cluster is surrounded by a median-centered ellipse and labeled according to its functional annotation. ISG, IFN-stimulated gene. (**C**) Distinct transcriptional signatures within clusters of SI PLZF^+^CD4^+^ T cells. Heatmap of unsupervised hierarchical clustering of DE genes by indicated cluster. Labels indicate genes utilized to determine functional annotation. (**D**) Pathway enrichment analysis within clusters of SI PLZF^+^CD4^+^ T cells. Dot plot of selected Gene Ontology terms, Reactome Gene Sets, and Kyoto Encyclopedia of Genes and Genomes pathways enriched within genes DE in each cluster by Metascape analysis. Downregulated pathways are indicated with negative number of genes and outliers indicated with text overlay.

We previously demonstrated that PLZF^+^CD4^+^ T cells were a transcriptionally distinct population that accounted for the majority of memory T cells in the developing human SI (14). We showed that PLZF^+^CD4^+^ T cells were significantly more likely to produce Th1 cytokines (IFN-γ, TNF-α) than conventional memory CD4^+^ T cells, displaying a heightened effector function and a transcriptional signature enriched for T cell activation genes. To better understand their full effector potential, we sorted memory PLZF^+^CD4^+^ T cells on the basis of proxy markers (Va7.2^–^CD45RA^–^CCR7^–^CD161^+^IL18R^+^PD1^+^) to greater than 90% purity using our previously validated sorting strategy (14) (Supplemental Figure 1A; supplemental material available online with this article; https://doi.org/10.1172/jci.insight.164672DS1) and analyzed the population-level transcriptional response to short-term stimulation with PMA/ionomycin. In addition to Th1-associated transcripts such as *TNF*, *IFNG*, and *XCL2*, stimulated PLZF^+^CD4^+^ T cells upregulated numerous cytokine transcripts not typically produced by a single Th subset. These included *IL4* and *IL13*, as well as *IL22* and *LIF*, indicating a potentially heterogeneous T cell population composed of distinct effector subsets (Figure 1A). To better define these subpopulations, we performed single-cell RNA sequencing (scRNA-Seq) on purified PLZF^+^CD4^+^ T cells from 3 individual donors using the same validated sorting strategy as above (Supplemental Figure 1, A–D). After quality control, a total of 23,676 memory PLZF^+^CD4^+^ T cells were included in the postanalysis. Unsupervised clustering of transcriptional data identified 7 individual clusters of PLZF^+^CD4^+^ T cells (c0–c6) defined by distinct expression signatures (Figure 1, B and C, and Supplemental Figure 1E). Interindividual phenotypic variation resulted in uneven donor contribution to specific clusters, yet all 3 donors contributed cells to each of the 7 clusters (Supplemental Figure 1, F and G). A subset of Th1-like cells with high expression of *XCL1*, *XCL2*, *CCL5*, *IFNG*, and *CXCR3*, as well as activation (*CD38*, *CD44*, *4-1BB/TNFRSF9*, and *NR4A2*) and cytotoxicity (*GZMA*, *GZMK*, and *NCR3/NKp30*) markers was readily identified (Figure 1C and Supplemental Figure 1E). In line with a Th1-like profile, pathway analysis revealed enrichment of IL-12 signaling, T cell receptor (TCR) signaling, and NK cell–mediated cytotoxicity (Figure 1D). Transcripts associated with Th17 and/or Th22 cells (*CCR6*, *RORC*, *RORA*, *MAF*, and *IL23R*) marked a second subset with increased expression of IL-2 (Figure 1C and Supplemental Figure 1E), as well as pathway enrichment for both IL-12 and TGF-β signaling (Figure 1D). A Th2-like cluster of PLZF^+^CD4^+^ T cells was characterized by the lineage-defining transcription factor *GATA3* and increased expression of *IL32*, *IL16*, and *MIF*, as well as the TCR signaling regulators *CD5* and *CD6* (Figure 1C and Supplemental Figure 1E). Proliferating cells were identified based on higher expression of transcripts related to G_1_/S phase (*CDC45*, *PCNA*, *TYMS*) or G_2_M phase (*CDK1*, *TOP2A*) (Figure 1, C and D, and Supplemental Figure 1E) (17), consistent with our previous identification of a large proportion of proliferating memory CD4^+^ T cells in the prenatal SI (14). An ISG signature defined another subset of PLZF^+^CD4^+^ T cells, which included the higher expression of *DDX58*, *OAS1*, *MX1*, *IFIT1*, *RSAD2*, and *ISG15* (Figure 1C and Supplemental Figure 1E) and was specifically enriched for type I and type II IFN signaling (Figure 1D). The ISG signature has been proposed to represent an intermediate Th1 activation state (18) and has been associated with bystander activation of T cells (19), a defining feature of prenatal PLZF^+^CD4^+^ T cells. Pathway analysis indicated that cytokine signaling, T cell activation, and T cell differentiation pathways were enriched among most subsets, whereas TCR signaling was uniquely enriched in Th1-like cells (Figure 1D). In stark contrast, c0 harbored only 22 differentially upregulated genes and was void of cytokine and TCR signaling pathways and markers of lymphocyte activation and differentiation, indicating a resting or precursor cell state. Cluster 5, while lacking a distinct gene signature, displayed transcriptional overlap with that of the Th-17/Th22-like subset suggestive of an intermediate or transitional state (Figure 1C and Supplemental Figure 1E). Last, expression of *TNF* and *LIF* transcripts was evident across all PLZF^+^CD4^+^ T cells without differential expression between clusters (Supplemental Figure 1H). These distinct transcriptional signatures suggest the presence of different effector branches of prenatal PLZF^+^CD4^+^ T cells that parallel conventional Th subsets.

**Figure 2:**
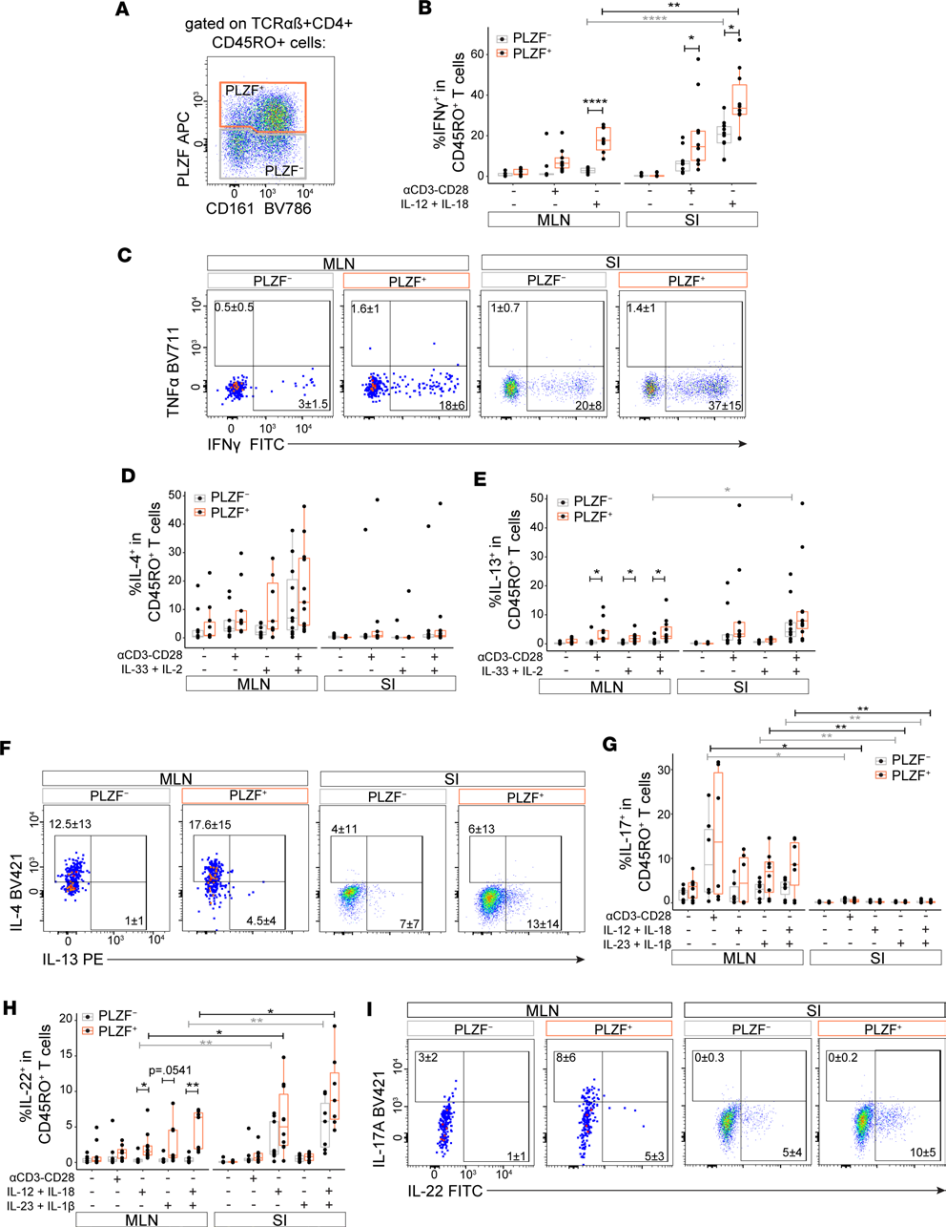
Prenatal PLZF^+^CD4^+^ T cells display spatially compartmentalized effector function. (**A**) Representative flow plot of the gating strategy used for the identification of cytokine-producing cells within memory PLZF^+^ and PLZF^–^ CD4^+^ T cells in the prenatal SI and MLNs. All PLZF^+^ and PLZF^–^ populations were pregated on TCRαβ^+^CD4^+^CD45RO^+^ T cells. (**B**–**I**) Distinct patterns of cytokine production within memory CD4^+^ T cells from the prenatal SI and MLNs. Quantification (**B**, **D**, **E**, **G**, and **H**) and representative flow plots (**C**, **F**, and **I**) of indicated intracellular cytokine staining after stimulation within PLZF^–^ (gray) and PLZF^+^ (orange) CD45RO^+^CD4^+^ T cells. Cells were stimulated as indicated for 16 hours, and Brefeldin A was added in the last 4 hours. Flow plots indicate representative intracellular cytokine staining following stimulation with (**C**) IL-12 + IL-18, (**F**) αCD3/CD28 monoclonal antibodies (mAbs) + IL-33 + IL-2, and (**I**) IL-23 + IL-1β + IL-12 + IL-18. Circles represent individual donors. Paired ANOVA with Tukey’s multiple-comparison test (**B**, **D**, **E**, **G**, and **H**). *P < 0.05, **P < 0.01, ****P < 0.0001. Gray and black asterisks and brackets denote comparison between PLZF^–^ and PLZF^+^ CD4^+^ T cell subsets, respectively. Large dots were used for improved visualization of small memory CD4^+^ T cell numbers within the MLNs. Numbers in flow cytometry plots represent the mean frequency of gated populations ± SD.

We next sought to verify cytokine production from our predicted Th-like subsets of memory PLZF^+^CD4^+^ T cells. We previously showed that PLZF^+^CD4^+^ T cells responded to TCR-mediated as well as to bystander (cytokine mediated) activation (14). A role for antigen-independent activation of CD8^+^ T cells has been described in the setting of inflammatory and/or infectious pathologies (20–22); less is known about bystander activation of human CD4^+^ T cells. To assess the human prenatal Th-like effector program, we stimulated memory CD4^+^ T cells from the SI and MLNs with anti-CD3/CD28 (αCD3/CD28) monoclonal antibodies and/or a combination of cytokines reported to elicit specific Th-type bystander responses (23). Consistent with our previous findings (14), we observed significantly higher frequencies of IFN-γ–producing cells within PLZF^+^ compared with PLZF^–^ CD45RO^+^CD4^+^ T cells (Figure 2, A–C). A similarly higher trend in the proportion of TNF-α^+^PLZF^+^CD45RO^+^CD4^+^ T cells was evident at an earlier time point, though this did not reach statistical significance (Supplemental Figure 2, A and B). Further, the combination of TCR and cytokine stimulation was synergistic for IFN-γ production among PLZF^+^CD4^+^ T cells (Supplemental Figure 2C). Differences in IFN-γ production were also evident by tissue, with significantly higher proportions of IFN-γ^+^ cells within both PLZF^+^ and PLZF^–^ subsets of SI memory CD4^+^ T cells compared with those of the MLN (Figure 2, B and C). Consistent with a Th2-like program, the proportion of IL-13–producing cells in the MLN was higher among PLZF^+^ than PLZF^–^ CD45RO^+^CD4^+^ T cells, whereas the proportions of IL-4^+^ cells were similar (Figure 2, D–F). However, IL-4 production was mostly absent from the SI, suggesting Th2-like cells in the prenatal intestine are primarily IL-13–producing cells with minimal capacity for IL-4 production. Although RORγt^+^PLZF^+^CD4^+^ T cells were present in both the MLN and the SI (Supplemental Figure 2D), there was a striking segregation of Th17- and Th22-like effector function between tissues (Figure 2, G–I). The proportion of IL-17^+^ cells was similar between PLZF^+^ and PLZF^–^CD45RO^+^CD4^+^ T cells in the MLNs, yet production of IL-17 was essentially absent from CD4^+^ T cells of the SI (Figure 2, G and I). Conversely, production of IL-22 was significantly higher among memory CD4^+^ T cells of the SI as compared with the MLNs and was elicited almost exclusively in response to bystander activation. Further, IL-22–producing cells were significantly more abundant within PLZF^+^ compared with PLZF^–^CD45RO^+^CD4^+^ T cells in the MLNs (Figure 2, H and I). These findings indicate the presence of functionally heterogeneous populations of prenatal PLZF^+^CD4^+^ T cells that correlate with the identification of distinct Th1-, Th2-, Th17-, and Th22-like transcriptional programs and reveal a striking segregation of effector function between the prenatal human MLNs and SI.

### IL-7 and TGF-β reciprocally regulate the expansion of prenatal naive PLZF^+^CD4^+^ T cells.

**Figure 3:**
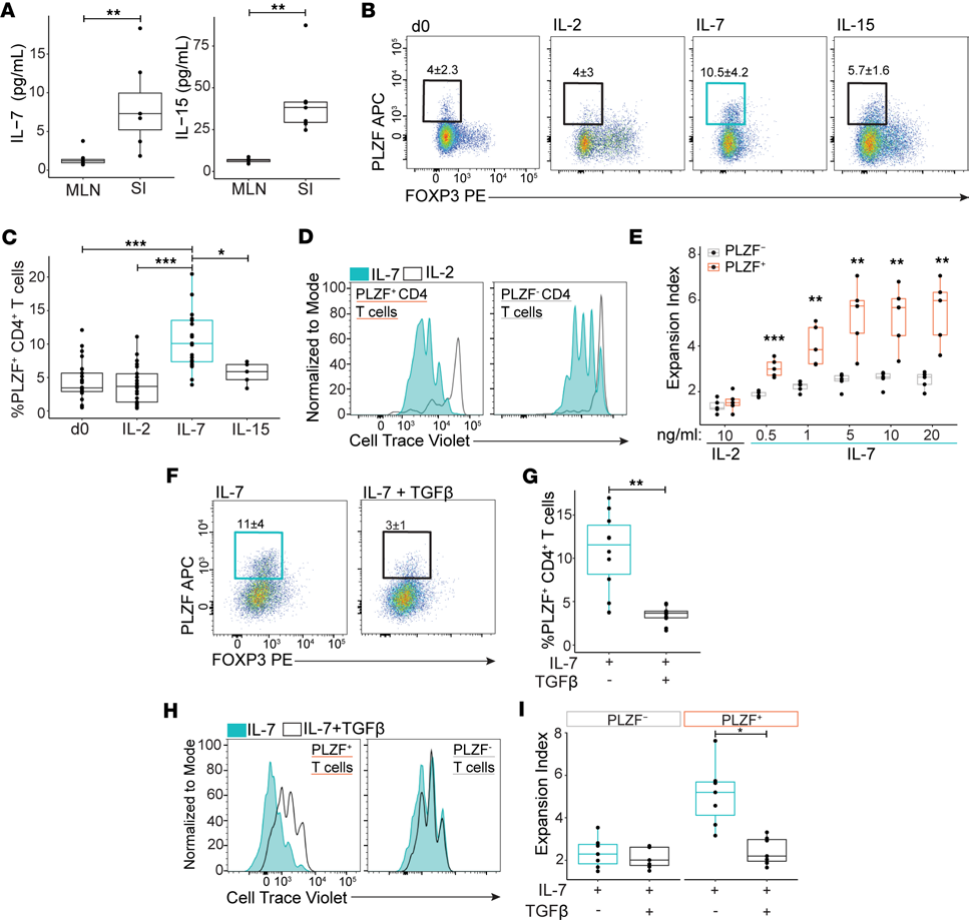
IL-7 and TGF-β reciprocally regulate the expansion of prenatal PLZF^+^CD4^+^ T cells. (**A**) Whole prenatal tissue concentrations of IL-7 and IL-15 measured by cytokine bead array identify higher cytokine levels in the SI compared with the MLNs (*n* = 6). (**B**–**E**) Specific accumulation of PLZF^+^CD4^+^ T cells in response to IL-7. (**B**) Representative flow plots and (**C**) frequencies of PLZF^+^CD4^+^ T cells derived from naive CD4^+^ T cells before initiation of culture (d0) and after 6 days of culture in the presence of indicated cytokines (10 ng/mL). (**D**) Representative histograms of Cell Trace Violet (CTV) dilution and (**E**) expansion indices after 6 days of culture depict the enhanced IL-7–driven proliferation of PLZF^+^CD4^+^ T cells. (**F**) Representative flow plots and (**G**) proportions of PLZF^+^CD4^+^ T cells indicate that addition of TGF-β (10 ng/mL) inhibits their accumulation after 6 days of culture in the presence of IL-7. (**H**) Representative histograms of CTV dilution and (**I**) expansion indices indicate the specific TGF-β–mediated inhibition of IL-7–driven proliferation within the PLZF^+^ subset of CD4^+^ T cells. Circles represent individual donors. Wilcoxon’s signed rank test (**A**, **G**, and **I**) and paired ANOVA with Tukey’s multiple-comparison test (**C** and **E**). *P < 0.05, **P < 0.01, ***P < 0.001. Numbers in flow cytometry plots represent the mean frequency of gated populations ± SD.

Based on the intestinal accumulation of PLZF^+^CD4^+^ T cells (14) and their enrichment for cytokine signaling pathways (Figure 1D), we hypothesized that differences in cytokine levels within tissues might contribute to their disparate distribution. Homeostatic cytokines such as IL-7 are systemically elevated in the setting of lymphopenia (24), yet we showed that tissue levels of IL-7 were distinct. We measured significantly higher levels of IL-7 and IL-15 in the prenatal SI as compared with the MLNs, while IL-2 levels were largely undetectable (Figure 3A and Supplemental Figure 3A). To examine the influence of these cytokines on the maturation and differentiation of CD4^+^ T cells, we obtained naive CD4^+^ T cells from mature CD1α^–^CD4^+^TCRαβ^+^ single-positive thymocytes to exclude prior exposure to peripheral homeostatic signals. We demonstrated a significant accumulation of PLZF^+^CD4^+^ T cells in response to IL-7 stimulation in comparison with prestimulation (d0) frequencies, an effect that was not evident in response to IL-2 or IL-15 (Figure 3, B and C). To define the cellular source of IL-7 in the prenatal SI, we measured protein levels within intestinal epithelial cells (IECs), as well as within hematopoietic cells and nonhematopoietic cells of the lamina propria. Consistent with previous reports (25, 26), we found that IL-7 protein was produced primarily within IECs (Supplemental Figure 3, B and C). We previously demonstrated that naive PLZF^+^CD4^+^ T cells have a 3-fold higher rate of ex vivo proliferation as compared with their conventional counterparts (14). We now show that PLZF^+^CD4^+^ T cells proliferate significantly more than their PLZF^–^ counterparts in response to IL-7 in a dose-dependent manner (Figure 3, D and E), which likely contributes to their accumulation.

Murine studies identify a correlation between surface expression of CD5, a marker of TCR signal strength on naive CD4^+^ and CD8^+^ T cells, and the intensity of lymphopenia-induced proliferation (27, 28). Thymic naive PLZF^+^CD4^+^ T cells expressed higher levels of CD5 as compared with their PLZF^–^ counterparts (Supplemental Figure 3, D and E), suggesting that the enhanced response to IL-7 may be set during thymic selection (29). It seemed plausible that the accumulation of PLZF^+^CD4^+^ T cells in response to IL-7 in the absence of TCR activation signals was due to the expansion of a thymus-derived population. To test this, we devised a new sorting strategy to enrich and deplete the native population of thymic naive PLZF^+^CD4^+^ T cells, as these could not be identified with the same proxy markers as those used on memory CD4^+^ T cells from the SI (Supplemental Figure 4, A and B). We show that the accumulation of PLZF^+^CD4^+^ T cells was dependent on the starting population, such that the higher the starting proportion of these cells, the more they accumulated in response to IL-7. Yet this relationship was not linear (Supplemental Figure 4, C and D). These data indicate that the accumulation of PLZF^+^CD4^+^ T cells in response to IL-7 is likely driven by their enhanced proliferation in comparison with naive PLZF^–^CD4^+^ T cells.

While the intestinal accumulation of PLZF^+^CD4^+^ T cells is associated with higher tissue levels of IL-7, the frequencies of FoxP3^+^ Treg cells are reciprocally increased in the prenatal MLNs (13, 30) and correlate with elevated levels of TGF-β that contributes to their generation (31). We thus postulated that TGF-β would inhibit the accumulation of PLZF^+^CD4^+^ T cells. Indeed, we found that TGF-β significantly dampened the accumulation of PLZF^+^CD4^+^ T cells in response to IL-7 by specifically inhibiting their proliferation relative to their PLZF counterparts (Figure 3, F–I). These data indicate that differences in steady-state cytokine levels within the prenatal SI and MLNs may contribute to the tissue distribution of PLZF^+^CD4^+^ T cells.

### Differences in IL-7 signaling contribute to the preferential expansion of PLZF^+^CD4^+^ T cells.

**Figure 4:**
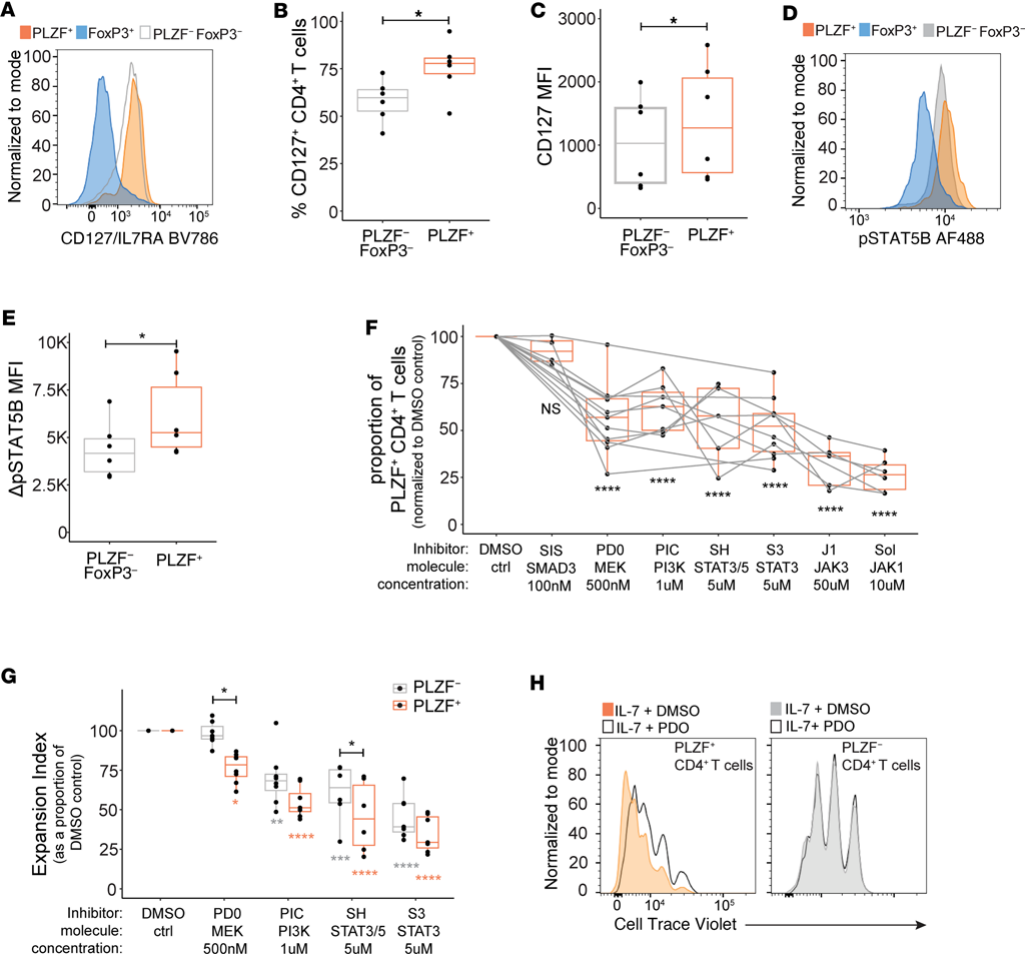
Differences in IL-7 signaling contribute to the preferential expansion of PLZF^+^CD4^+^ T cells. (**A**) Representative histograms, (**B**) frequencies, and (**C**) mean fluorescence intensity (MFI) of IL-7R expression within subsets of naive CD4^+^ T cells (*n* = 6). (**D**) Representative histograms and (**E**) normalized MFI of phosphorylated STAT5B (pSTAT5B) within subsets of naive CD4^+^ T cells after 30 minutes of stimulation with IL-7 (*n* = 6). (**F**) Normalized proportions of PLZF^+^CD4^+^ T cells relative to DMSO control after 6 days of stimulation with IL-7 in the presence of indicated signaling inhibitors. Inhibitors used: SIS (SIS3, SMAD3), PD0 (PD0325901, MEK), PIC (pictilisib, PI3Kα/δ), SH (SH-4-54, STAT3 and STAT5), S3 (STAT3-IN-1, STAT3), J1 (JANEX-1, JAK3), and Sol (solcitinib, JAK1). Gray lines connect values derived from the same donor. (**G**) Normalized expansion indices relative to DMSO control within subsets of CD4^+^ T cells after 6 days of stimulation with IL-7 in the presence of the indicated signaling inhibitors and (**H**) representative histograms of CTV dilution within indicated populations of naive CD4^+^ T cells after treatment with IL-7 and PD0 depict the selective sensitivity of PLZF^+^CD4^+^ T cells to MEK/ERK signaling. Circles represent individual donors. Wilcoxon’s signed rank test (**B**, **C**, and **E**) and paired ANOVA with Tukey’s multiple-comparison test (**F** and **G**). *P < 0.05, **P < 0.01, ***P < 0.001, ****P < 0.0001.

IL-7 is known to drive the homeostatic expansion of human naive CD4^+^ T cells during early life lymphopenia (32), which led us to examine whether differences in IL-7 receptor (IL-7R) signaling might contribute to the preferential expansion of prenatal naive PLZF^+^CD4^+^ T cells. The binding of IL-7 to the IL-7R activates JAK1 and JAK3/STAT5 signaling as well as STAT-independent signal transduction pathways, including PI3K/AKT and MEK/ERK (33). Expression of IL-7R was evident in the majority of naive FoxP3^–^CD4^+^ T cells and was significantly more prevalent among PLZF^+^CD4^+^ T cells, whereas expression of IL-2RA, IL-15RA, and IL-15RB did not differ from those of their PLZF^–^counterparts (Figure 4, A–C, and Supplemental Figure 5, A–F). In line with cytokine receptor expression, induction of STAT5B phosphorylation (p-STAT5B) was specifically higher in response to IL-7, but not IL-2 or IL-15, in naive PLZF^+^ compared with PLZF^–^ CD4^+^ T cells (Figure 4, D and E, and Supplemental Figure 5, G and H). Next, we examined the influence of small molecule inhibitors of specific IL-7R signaling pathways on the accumulation and proliferation of PLZF^+^CD4^+^ T cells. As expected, inhibition of the upstream signaling mediators JAK1 and JAK3 resulted in the most striking loss of IL-7–driven accumulation of PLZF^+^CD4^+^ T cells, an effect likely related to the near-complete inhibition of proliferation (Figure 4F and Supplemental Figure 5, I and J). A similar dependency on STAT5 and STAT3 signaling was also evident. However, the combined STAT3 and STAT5 inhibitor resulted in a more significant decrease in the expansion of PLZF^+^ compared with PLZF^–^ CD4^+^ T cells, whereas the response to the STAT3-specific inhibitor was similar between the two (Figure 4, F and G, and Supplemental Figure 5, K and L). These data are in line with the enhanced induction of p-STAT5B in PLZF^+^CD4^+^ T cells as compared with the PLZF^–^ subset (Figure 4, D and E). We additionally observed significant sensitivity to STAT-independent IL-7R signaling. Inhibition of either MEK/ERK or PI3K significantly dampened the IL-7–driven accumulation of PLZF^+^CD4^+^ T cells, whereas inhibition of SMAD3 had no effect (Figure 4F). Interruption of MEK/ERK signaling selectively inhibited the proliferation of PLZF^+^CD4^+^ T cells, whereas dependence on PI3K signaling was similar between PLZF^+^ and PLZF^–^ CD4^+^ T cells (Figure 4, G and H, and Supplemental Figure 5M). In sum, these data indicate a heightened sensitivity to STAT5 and a selective sensitivity to MEK/ERK signaling in PLZF^+^CD4^+^ T cells relative to their PLZF^–^ counterparts, which likely contributes to their preferential expansion and accumulation in response to IL-7.

### Prenatal PLZF^+^CD4^+^ T cells are MHC class II restricted, and TCR signaling does not interfere with IL-7–driven expansion.

**Figure 5:**
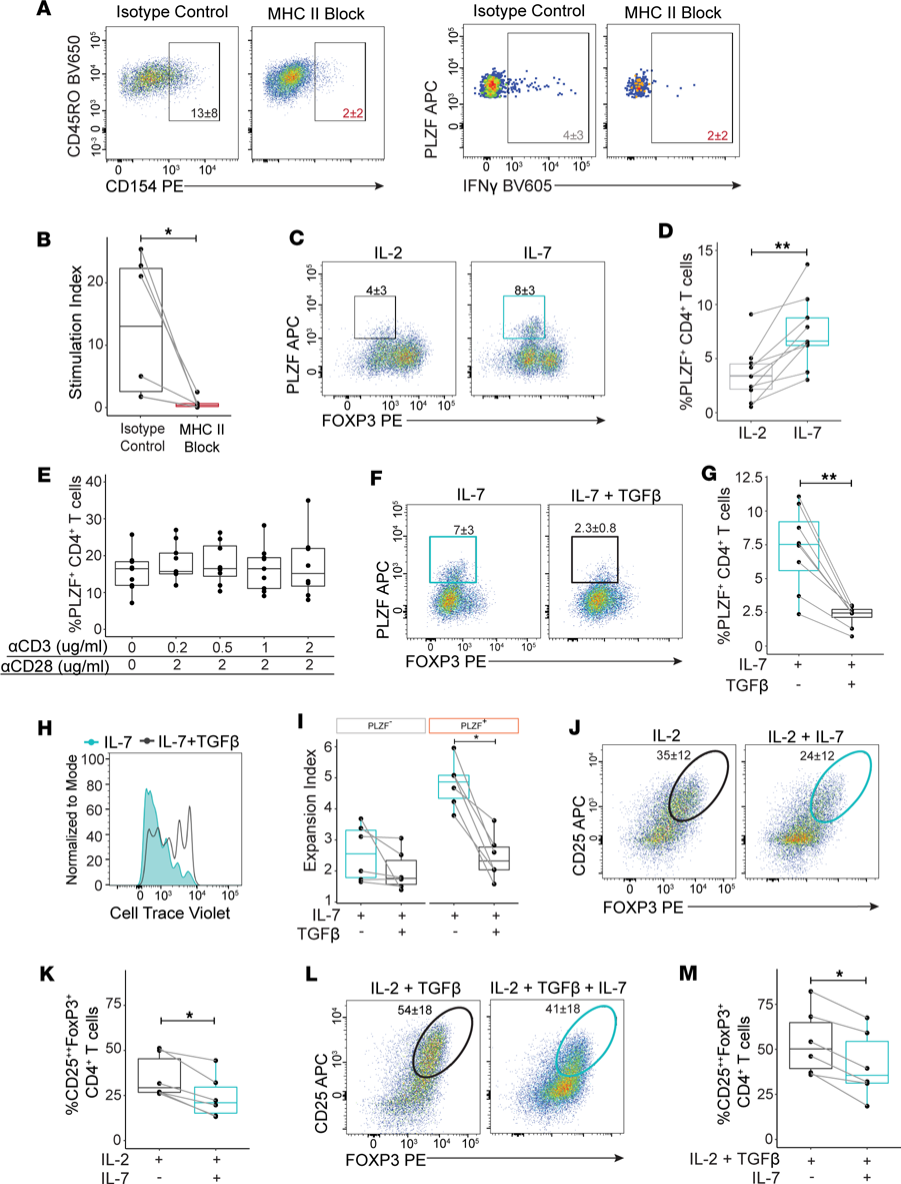
IL-7 and TGF-β sustain reciprocal regulation of MHC class II–restricted PLZF^+^CD4^+^ naive T cells in the presence of TCR signaling. (**A** and **B**) Blocking of MHC class II–TCR interactions inhibits the activation of PLZF^+^CD4^+^ T cells. (**A**) Representative flow plots of intracellular CD40L/CD154 expression within SI PLZF^+^CD4^+^ T cells (top) and IFN-γ production within CD154^+^PLZF^+^CD4^+^ T cells (bottom) cocultured with allogeneic adult CD14^+^ APCs in the presence of a pan–MHC-II blocking antibody (αHLA DR-DP-DQ) or isotype control. (**B**) Stimulation index, calculated as the percentage of CD154^+^IFN-γ^+^ within PLZF^+^CD4^+^ T cells after 16 hours of coculture with allogeneic adult CD14^+^ APCs in the presence of a pan MHC II blocking antibody (αHLA DR-DP-DQ) compared with isotype control (IgG_2_). (**C**–**E**) TCR signaling does not interfere with the IL-7–driven accumulation of PLZF^+^CD4^+^ T cells. (**C**) Representative flow plots and (**D**) frequencies of PLZF^+^CD4^+^ T cells derived from naive CD4^+^ T cells stimulated with αCD3/CD28 in the presence of IL-2 or IL-7 for 6 days. Gray lines connect values derived from the same donor. (**E**) Proportions of PLZF^+^CD4^+^ T cells stimulated with indicated concentrations of αCD3/CD28 in the presence of IL-7 for 12 days. (**F**) Representative flow plots and (**G**) proportions of PLZF^+^CD4^+^ T cells indicate that stimulation with αCD3/CD28 does not interfere with the TGF-β–mediated inhibition of their IL-7–driven accumulation. (**H**) Representative histograms of CTV dilution and (**I**) expansion indices within indicated CD4^+^ T cell subsets after 6 days of stimulation with αCD3/CD28 in the presence of IL-7 ± TGF-β. (**J** and **L**) Representative flow plots and (**K** and **M**) frequencies of CD25^hi^FoxP3^+^CD4^+^ T cells after 6 days of αCD3/CD28 stimulation in the presence of indicated cytokines. Circles represent individual donors. Wilcoxon’s signed rank test (**B**, **D**, **G**, **I**, **K**, and **M**) and paired ANOVA with Tukey’s multiple-comparison test (**E**). *P < 0.05, **P < 0.01. Numbers in flow cytometry plots represent the mean frequency of gated populations ± SD.

IL-7 can function independently of, or synergize with, TCR signaling to promote proliferation of naive CD4^+^ T cells (34). As prenatal PLZF^+^CD4^+^ T cells share functional and transcriptional attributes with both conventional and innate-like T cells (14), we next sought to determine whether PLZF^+^CD4^+^ T cell activation was mediated by recognition of MHC class II. We examined the effect of blocking MHC class II–TCR interactions on the activation of SI memory PLZF^+^CD4^+^ T cells during short-term exposure to allogeneic adult CD14^+^ antigen-presenting cells (APCs) ex vivo. We quantified the response by calculating a stimulation index, defined as the proportion of PLZF^+^CD4^+^ T cells that upregulated expression of the activation molecule CD154 (CD40L) multiplied by the proportion of IFN-γ–producing cells (35). We found that the stimulation index of PLZF^+^CD4^+^ T cells was significantly reduced in the presence of a pan–MHC class II blocking antibody in comparison with isotype control (Figure 5, A and B). Thus, consistent with the expression of a polyclonal TCR repertoire (14), prenatal PLZF^+^CD4^+^ T cells recognized antigen in the context of MHC class II, suggesting a stronger similarity to conventional CD4^+^ T cells than to innate-like T cells.

We next evaluated the influence of TCR activation on the expansion of PLZF^+^CD4^+^ T cells in response to IL-7. Activation via αCD3/CD28 cross-linking did not affect their preferential accumulation in the presence of IL-7 (Figure 5, C and D), a finding consistent across varying levels of αCD3 stimulation (Figure 5E). We also excluded the possibility that low-level TCR signaling from T cell–T cell interactions (36) might contribute to the IL-7–driven accumulation of PLZF^+^CD4^+^ T cells as this was unchanged in the presence of a pan–MHC class II blocking antibody (Supplemental Figure 6A). Further, TCR engagement had no effect on the ability of TGF-β to inhibit the IL-7–driven accumulation and proliferation of PLZF^+^CD4^+^ T cells (Figure 5, F–I). As prenatal naive T cells are known to preferentially differentiate into Treg cells upon encounter with antigen, we also examined the effect of IL-7 on this process (31). Whether naive CD4^+^ T cells were activated in the absence of Th-skewing conditions (Th0; IL-2 alone) or under Treg-skewing conditions (IL-2 + TGF-β), exposure to IL-7 significantly inhibited the generation of Treg cells (Figure 5, J–M). These data indicate that IL-7 and TGF-β reciprocally regulate the expansion of PLZF^+^CD4^+^ T cells and the differentiation of Treg cells and may contribute to the divergent tissue distribution of these prenatal T cell subsets.

### IL-7 is sufficient to induce the emergence of memory phenotype PLZF^+^CD4^+^ T cells.

**Figure 6:**
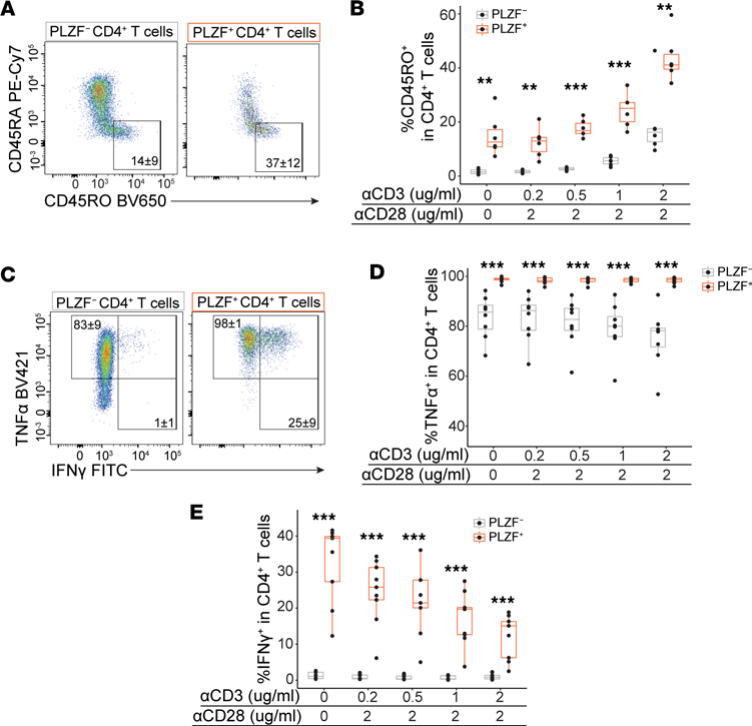
IL-7 is sufficient to promote the emergence of memory phenotype PLZF^+^CD4^+^ T cells. (**A**) Representative flow plots and (**B**) frequencies of CD45RO^+^ cells within PLZF^+^ and PLZF^–^ CD4^+^ T cells after 12 days of culture with IL-7 in the presence of indicated concentrations of αCD3/CD28 stimulation. (**C**) Representative flow plots of intracellular cytokine staining and (**D** and **E**) frequencies of (**D**) TNF-α^+^ and (**E**) IFN-γ^+^ cells within subsets of CD4^+^ T cells following restimulation with PMA/ionomycin after 12 days of culture with IL-7 and indicated concentrations of αCD3/CD28 stimulation. Circles represent individual donors. Paired ANOVA with Tukey’s multiple-comparison test (**B**, **D**, and **E**). **P < 0.01, ***P < 0.001. Numbers in flow cytometry plots represent the mean frequency ± SD of gated populations.

As long-term polyclonal stimulation of human naive T cells in vitro results in the generation of CD45RO^+^ memory cells (37), we next analyzed the induction of a memory phenotype in PLZF^+^CD4^+^ T cells exposed to IL-7 in the presence of varying levels of αCD3/CD28 stimulation. Consistent with previous data, we demonstrated the emergence of CD45RO^+^ memory cells after prolonged polyclonal stimulation of prenatal naive CD4^+^ T cells, albeit the frequencies of these were significantly higher among PLZF^+^ as compared to PLZF^–^ CD4^+^ T cells across conditions (Figure 6, A and B). Moreover, there was selective acquisition of CD45RO expression with the PLZF^+^ subset of CD4^+^ T cells upon exposure to IL-7 alone, and the proportions of CD45RO^+^ cells were comparable to those generated in response to the addition of anti-CD3/CD28 stimulation (Figure 6B). We further showed that the IL-7–driven acquisition of CD45RO expression in PLZF^+^CD4^+^ T cells was not significantly affected by the blockade of low-level MHC/TCR signaling from T cell–T cell interactions (Supplemental Figure 7A). To determine whether the expression of CD45RO corresponded with the acquisition of effector potential, we stimulated CD4^+^ T cells with PMA/ionomycin after prolonged culture in the presence of IL-7 and increasing levels of TCR activation. While the potential for TNF-α production was high among all prenatal CD4^+^ T cells, the proportion of TNF-α^+^ cells was consistently higher in PLZF^+^ as compared with PLZF^–^ CD4^+^ T cells and did not differ across levels of αCD3 activation (Figure 6, C and D). Conversely, IFN-γ production was nearly absent from PLZF^–^CD4^+^ T cells, and the proportion of IFN-γ^+^PLZF^+^ T cells was inversely related to the strength of TCR stimulation (Figure 6, C and E). These results indicate that IL-7 is sufficient to induce the emergence of memory phenotype PLZF^+^CD4^+^ T cells with the potential for IFN-γ production and identify a human counterpart to murine memory phenotype CD4^+^ T cells.

### IL-7 modulates the effector maturation of prenatal PLZF^+^CD4^+^ T cells.

**Figure 7:**
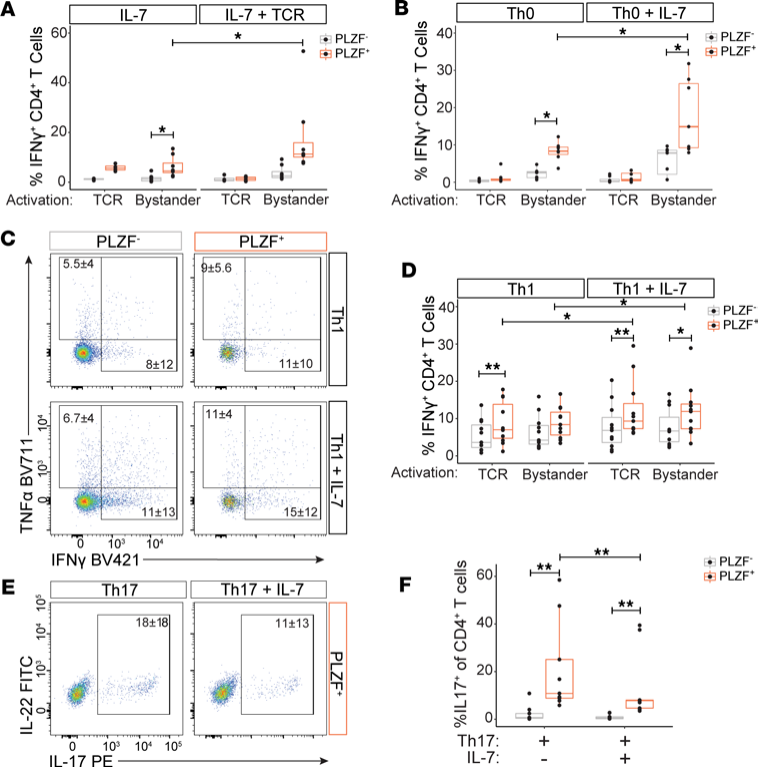
IL-7 modulates the effector maturation of prenatal PLZF^+^CD4^+^ T cells. Functional studies following a 2-step in vitro culture in which naive CD4^+^ T cells were first expanded in the presence of IL-7 alone, followed by 7 days of maturation in the indicated conditions: IL-7 alone, IL-7 + TCR (stimulated with αCD3/CD28 mAbs), Th0 (TCR + IL-2), Th1 (TCR + IL-12), Th17 (TCR + IL-23 + IL-1β). (**A**–**D**) IL-7 enhances the emergence of IFN-γ^+^PLZF^+^CD4^+^ T cells. (**A**, **B**, and **D**) Proportion of IFN-γ^+^ cells within CD4^+^ T cell subsets exposed to the indicated differentiation conditions and following 24-hour restimulation with αCD3/CD28 (TCR) or IL-12/IL-18 (Bystander). (**C**) Representative flow plots of intracellular cytokine staining within subsets of CD4^+^ T cells differentiated under indicated conditions and following 24-hour restimulation with αCD3/CD28. (**E** and **F**) IL-7 dampens the emergence of IL-17^+^PLZF^+^CD4^+^ T cells. (**E**) Representative flow plots of intracellular cytokine staining and (**F**) proportions of IL-17^+^ cells within indicated CD4^+^ T cell subsets matured under Th17-skewing conditions and following PMA/ionomycin stimulation. Circles represent individual donors. Wilcoxon’s signed rank test to compare PLZF^+^ to PLZF^–^ fractions and paired stimulations across maturation conditions (**A**, **B**, **D**, and **F**). *P < 0.05, **P < 0.01. Numbers in flow cytometry plots represent the mean frequency of gated populations ± SD.

We next asked whether memory phenotype PLZF^+^CD4^+^ T cells generated in response to IL-7 alone, or in combination with TCR signaling, could produce cytokines in response to antigen receptor–mediated or bystander activation. To ensure sufficient cell numbers for functional interrogation, we first cultured naive CD4^+^ T cells in IL-7 for 6 days to promote the expansion of PLZF^+^CD4^+^ T cells and subsequently differentiated them in the presence of αCD3/CD28 and various combinations of cytokines (Supplemental Figure 8A). A subset of PLZF^+^CD4^+^ T cells produced IFN-γ in response to both TCR- and cytokine-mediated activation after prolonged culture in IL-7 alone, as well as TNF-α in response to TCR activation, while cytokine production was mostly absent from PLZF^–^CD4^+^ T cells (Figure 7A and Supplemental Figure 8B). Differentiation of naive CD4^+^ T cells in the absence of lineage-skewing conditions (Th0) did not generate IFN-γ–producing cells in response to TCR-mediated activation yet resulted in significantly higher proportions of PLZF^+^CD4^+^ T cells capable of IFN-γ production in response to bystander activation as compared with their PLZF^–^ counterparts (Figure 7B). Moreover, the proportion of bystander-responsive IFN-γ^+^PLZF^+^CD4^+^ T cells was further enhanced by the addition of IL-7, whereas TNF-α production was unaffected (Figure 7B and Supplemental Figure 8C). Whole tissue levels of IL-12p70, the bioactive form of IL-12 required for Th1 differentiation, were comparable between the prenatal SI and the MLNs, indicating that low-level, homeostatic production could contribute to the generation of effector T cells (Supplemental Figure 8D). Although Th1 differentiation induced the generation of TCR-responsive IFN-γ– and TNF-α–producing CD4^+^ T cells, the proportion of these was consistently higher among PLZF^+^CD4^+^ T cells. Further, the addition of IL-7 to Th1 differentiation conditions specifically enhanced the proportion of IFN-γ–producing PLZF^+^CD4^+^ T cells in response to both TCR-mediated and bystander activation yet had no effect on their PLZF^–^ counterparts (Figure 7, C and D, and Supplemental Figure 8E).

Despite the observed compartmentalization of IL-17–producing CD4^+^ T cells to the MLNs (Figure 2G), tissue levels of IL-1β and IL-23 required for human Th17 differentiation were significantly higher in the SI (Supplemental Figure 8F). The capacity for IL-17 production following naive CD4^+^ T cell differentiation in the presence of IL-1β and IL-23 was restricted to the PLZF^+^ subset of CD4^+^ T cells and was unaffected by the addition of IL-6 or IL-6 plus TGF-β (Supplemental Figure 8G). However, the addition of IL-7 to Th17 differentiation conditions significantly dampened the potential for IL-17 production among PLZF^+^CD4^+^ T cells (Figure 7, E and F). Together, these data reveal a role for IL-7 in modulating effector maturation of prenatal PLZF^+^CD4^+^ T cells, where IL-7 reciprocally enhances the production of IFN-γ and inhibits the potential for IL-17 production. This divergent maturation in response to IL-7 is likely a contributing factor to the observed compartmentalization of prenatal CD4^+^ T cell effector function between the prenatal SI and the MLNs.

## Discussion

This study provides evidence for the emergence of human protective adaptive immunity with anatomically compartmentalized effector function during the second trimester of human gestation that parallels conventional Th1, Th2, Th17, and Th22 cells. Through transcriptional analysis, protein validation, and in vitro culture of primary human cells, we identified a critical role for IL-7 in the tissue-specific accumulation and effector maturation of human prenatal PLZF^+^CD4^+^ T cells that likely contributes to their intestinal segregation. These results contribute to the current shift in our understanding of human prenatal immunity from one of functional immaturity to that of a specialized adaptation to meet the concurrent demands for prenatal tolerance and rapid postnatal protection.

Our current work demonstrates a central role for IL-7 in the emergence of human prenatal memory phenotype PLZF^+^CD4^+^ T cells. These results are congruent with murine studies that identified the generation of memory phenotype CD4^+^ T cells with rapid effector function in the setting of physiologic neonatal lymphopenia (9). Early-life specialization is evident in neonatal CD8^+^ T cells, which preferentially develop into virtual memory T cells and play a role in the early response to infection in mice (10). Little is known about the origin and function of memory phenotype CD4^+^ T cells during early life in humans. The expansion of the early-life peripheral human naive T cell pool is driven primarily by the homeostatic proliferation and survival of naive CD4^+^ T cells (38–40), a process dependent on IL-7 (32, 41). Systemic levels of IL-7 are increased in lymphopenic hosts (24), with the highest levels reported in lymphoid organs such as the thymus and lymph nodes (42). Notably, we detected significantly higher levels of IL-7 protein in the human prenatal SI compared with the MLNs (Figure 3A), which mirrors the preferential accumulation of memory PLZF^+^CD4^+^ T cells with enhanced Th1-like effector function in the SI over the MLNs. Our data demonstrate that IL-7 is sufficient to drive both the expansion and maturation of prenatal naive PLZF^+^CD4^+^ T cells and identify the human analog of early-life memory phenotype CD4^+^ T cells.

In mice, the generation of memory phenotype CD4^+^ T cells depends on tonic, low-level signaling from TCR–MHC class II interactions primarily in response to self-antigens (8). Self-reactivity is evident among human prenatal CD4^+^ T cells (31), and we and others have also demonstrated that prenatal intestinal CD4^+^ T cells respond to microbial antigens (43, 44). Although blocking TCR–MHC class II interactions in the short term did not influence the proliferation or acquisition of memory phenotype of PLZF^+^CD4^+^ T cells in vitro, it is possible that TCR signals are required for their long-term survival in vivo (45). Future work will determine whether IL-7–expanded and ex vivo–isolated prenatal T cells are responsive to self- and/or foreign antigens.

T cell activation in response to cognate antigens remains one of the hallmark features of adaptive immunity. In early life, recognition of danger signals through antigen-independent activation may serve a rapid protective function at barrier surfaces. Similar to virtual memory CD8^+^ T cells, memory phenotype CD4^+^ T cells have been reported to provide early protection against pathogens (8, 46), suggesting a vital role in the setting of early-life immunity. In stark contrast to the mucosal predominance of Th17 cells in mice (47) and human adults (48), prenatal IL-17–producing CD4^+^ T cells segregated to the MLNs and were absent from the SI, which instead contained higher frequencies of IL-22–producing cells. The anatomic segregation of steady-state prenatal IL-17– and IL-22–producing CD4^+^ T cells to the MLNs and SI, respectively, may serve to limit perinatal inflammation upon encounter with swallowed luminal antigens or inflammatory mediators while promoting the development and maintenance of epithelial barrier function (49). Production of IL-22 among CD4^+^ T cells was elicited almost exclusively in response to bystander activation, which suggests a memory phenotype origin, and more work is needed to identify the cues that drive their effector maturation.

The heterogeneity and spatial segregation of effector function posits that PLZF^+^CD4^+^ T cells are composed of a combination of lymphopenia-induced, memory phenotype cells as well as antigen-experienced memory T cells. Although PLZF expression is evident during thymic development, neither mature CD4^+^ thymocytes nor SI naive CD4^+^ T cells were capable of IFN-γ production, indicating that, unlike mucosal associated invariant T or NK T cells, additional steps were required for effector maturation (14). We show that PLZF^+^CD4^+^ T cells recognized antigen in the context of MHC class II, which, coupled with a polyclonal TCR repertoire (14), indicates substantial overlap with conventional CD4^+^ T cells, including the presence of distinct Th-like subsets. Migratory APCs capable of sensing pathogen and stimulating T cells are evident by the second trimester of gestation (50), and antigen-specific immunity to prenatal pathogen exposure is well documented (51), indicating that Th differentiation is likely occurring in utero. We detected low yet consistent levels of Th-skewing cytokines within the prenatal SI, which consistently elicited a more robust effector response from PLZF^+^CD4^+^ T cells in vitro. Moreover, our data identify a role for IL-7 in the modulation of Th1- and Th17-like differentiation among PLZF^+^CD4^+^ T cells and provide a plausible mechanism for the tissue-specific segregation of prenatal effector function.

Beyond extending our understanding of human immune ontogeny, an improved understanding of the composition, spatial distribution, and functional responses of prenatal T cells is foundational to the development of early-life immunomodulatory interventions with potentially lifelong consequences on immune health.

## Methods

### Cell isolation.

PBMCs from adult blood were isolated by Ficoll-Histopaque (MilliporeSigma) gradient centrifugation and cryopreserved in freezing medium (90% FBS + 10% DMSO; ATCC) for batch analysis. Prenatal organs were processed within 2 hours of collection as previously described (14). Briefly, the MLNs and SI were dissected, washed, and cut into 1 cm fragments. Mucus from the SI was removed by 3 × 20-minute DTT washes, and the SI epithelial cells (IECs) were removed by 3 × 20-minute EDTA washes at 37°C. The MLNs and SI were digested in collagenase IV (Life Technologies) and DNase (Roche) for 45 minutes at 37°C, then filtered, and lymphocytes were enriched by 20/40/80 Percoll (GE Healthcare, now Cytiva) gradient centrifugation. SI and MLN CD4^+^ T cells were further enriched by negative selection using the EasySep Human CD4 T Cell Isolation Kit (STEMCELL Technologies). In select experiments (Supplemental Figure 2, A–C) CD4^+^ T cells were isolated as described above from cryopreserved MLN and SI tissue fragments. Thymocyte single-cell suspensions were obtained by pressing minced tissue through a 70 μm strainer (Corning). Primary naive CD4^+^ T cells were obtained from mature CD4–single-positive (CD4 SP) thymocytes. Mature CD1a^–^CD4^+^TCRαβ^+^ SP thymocytes were obtained by negative selection using the EasySep Biotin Positive Selection Kit (STEMCELL Technologies) with the modification of an added wash step after staining of whole thymocytes with mAbs for CD34, CD56, CD14, CD11c, CD19, CD1a, and CD8a as previously described (52). Viability was measured with trypan blue (MilliporeSigma).

### Antibodies and flow cytometry.

Cells were incubated in 2% FCS in PBS with 1 mM EDTA with human Fc block (STEMCELL Technologies) and stained with Aqua LIVE/DEAD Fixable Dead Cell Stain Kit (Invitrogen) and fluorochrome-conjugated antibodies against surface markers. Intracellular protein and cytokine staining was performed using the Foxp3/Transcription Factor Staining Buffer Set (Tonbo Biosciences). Mouse and rat anti-human mAbs used in this study included IFN-γ FITC (clone 25723.11, BD Biosciences, catalog 340449), TCRαβ Percp 710 (clone IP26, Invitrogen, catalog 46-9986-42), TCRγδ Pe-CF594 (clone B1, BD Biosciences, catalog 562511), CD45RA PE-Cy7 (clone HI100, BD Biosciences, catalog 560675), CD4 APC-H7 (clone L200, BD Biosciences, catalog 560837), PLZF APC (clone 6318100, R&D Systems, catalog IC2944A), TCR Va7.2 BV605 (clone 3C10, BioLegend, catalog 351720), CD45RO BV650 (clone UCHL1, BioLegend, catalog 304232), TNF-α BV711 (clone MAb11, BioLegend, catalog 502940), CD161 BV785 (clone HP-3G10, BioLegend, catalog 339930), CD45 BUV395 (clone HI30, BD Biosciences, catalog 563792), CD3 BUV737 (clone UCHT1, BD Biosciences, catalog 612750), CD8 APC-R700 (clone RPA-T8, BD Biosciences, catalog 565166), IL-22 FITC (clone 22URT1, Invitrogen, catalog 11-7229-42), IFN-γ BV421 (clone 4S.B3, BD Biosciences, catalog 564791), IL-2 BV711 (clone 5344.111, BD Biosciences, catalog 563946), IL-17A BV421 (clone BL168, BioLegend, catalog 512322), IFN-γ BV711 (clone 4S.B3, BioLegend, catalog 502539), IL-13 PE (clone JES10-5A2, BioLegend, catalog 501903), IL-4 BV421 (clone MP4-25D2, BioLegend, catalog 500826), Vα7.2 Biotin (clone 3C10, BioLegend, catalog 351724), Va2.4 Biotin (clone 6B11, Invitrogen, catalog 13-5806-82), Streptavidin APC-R700 (BD Biosciences, catalog 565144), CD154 PE (clone TRAP1, BD Biosciences, catalog 555700), TNF-α PECy7 (clone MAb11, BD Biosciences, catalog 557647), CTV BV421 (Thermo Fisher Scientific, catalog C34571), IFN-γ BV605 (clone 4S.B3, BioLegend, catalog 502536), CD8 BV711 (clone RPA-T8, BD Biosciences, catalog 563677), CD215 BV421 (clone JM7A4, BD Biosciences, catalog 747704), CD127 BV786 (clone HIL-7R-M21, BD Biosciences, catalog 563324), CD25 FITC (clone M-A251, BioLegend, catalog 356106), CD8 FITC (clone RPA-T8, BioLegend, catalog 301050), CD161 BV711 (clone DX12, BD Biosciences, catalog 563865), FoxP3 PE (clone PCH101, Invitrogen, catalog 12-4776-41), PD-1 BV605 (clone EH12.2H7, BioLegend, catalog 329924), CD8 PeCY7 (clone RPA-T8, BD Biosciences, catalog 557746), Streptavidin BV605 (BioLegend, catalog 405229), CCR7 BV421 (clone G043H7, BioLegend, catalog 353208), CD4 APC-Cy7 (clone RPA-T4, BioLegend, catalog 300517), and Aqua LD (Invitrogen, catalog L34957).

For phosphoflow staining, 1 million CD4^+^ SP thymocytes were stimulated with IL-7 at 5 ng/mL for 30 minutes at room temperature. The reaction was stopped by adding Fix/Perm and subsequently stained for p-STAT5B (pY694, BD Biosciences) in a 96-well plate using the Foxp3/Transcription Factor Staining Buffer Set. All data were acquired with an LSR/Fortessa Dual SORP flow cytometer (BD Biosciences) and analyzed with FlowJo V10.0.8 (TreeStar) software. Expansion index was calculated using FlowJo software according to the formula expansion index = (1 – PF)/(1 – Dil), where PF = fraction of the original population dividing at leads once during the culture period and Dil = percentage of cells in the final population that have divided.

### Bulk RNA-Seq.

T cells were isolated as described above from the intestine of 3 individual samples, and PLZF^+^CD4^+^TCRαβ^+^ T cells were sorted on proxy surface markers (Va7.2^–^, CD45RA^–^, CCR7^–^, CD161^+^, IL18R^+^, PD1^+^) using FACSAria2 SORP (BD Biosciences) as detailed in Supplemental Figure 1A and as previously described (14). Half the sorted cells were stimulated with 50 ng/mL PMA (Santa Cruz Biotechnology) and 5 μg/mL ionomycin (MilliporeSigma) for 3 hours at 37°C in 4% O_2_; the remaining cells were left unstimulated. RNA was extracted and purified with the Dynabeads mRNA DIRECT Purification Kit (Thermo Fisher Scientific). mRNA libraries were constructed using the Nugen/Nextera XT Library Prep Kit (Illumina), and 3 samples (3 donors) were sequenced on an Illumina HiSeq 4000 by the Functional Genomics Core Facility at UCSF. The reads from the Illumina HiSeq sequencer in fastq format were verified for quality control using the fastqc software package. Reads were aligned to the human genome (hg38) and read counts aggregated by gene using the Ensembl GRCh38.78 annotation using STAR (53). Transcriptional analysis of unstimulated prenatal PLZF^+^CD4^+^ T cells used for comparison was previously published (14). Differential gene expression analysis was performed on all genes with at least 10 reads with DESeq2, version 1.16.1 (54). Volcano plots were created with ggplot2 depicting DE genes (log_2_ fold-change > 0.5, FDR ≤ 0.05).

### ScRNA-Seq.

Single, live PLZF^+^CD4^+^TCRαβ^+^ T cells from the SI were sorted on proxy markers (Va7.2^–^, CD45RA^–^, CCR7^–^, CD161^+^, IL18R^+^, PD1^+^) using the same strategy as that for the purification of these cells for bulk RNA-Seq described above, as outlined in Supplemental Figure 1A, and as previously described (14). Postsort purity was more than 89% as determined by intracellular staining for PLZF. Single cells were captured by droplet-based microfluidics, then lysed, and sequencing libraries were prepared in a single bulk reaction following the 10x Genomics protocol. Transcripts were sequenced using a HiSeq4000 System (Illumina). FASTQ files from each multiplexed capture library were mapped to a custom reference containing GRCh19 using the Cell Ranger (v2.0.0) (10x Genomics) count function. After demultiplexing cells into samples, Seurat (v3.1.5) was used to perform quality control filtering of cells. Cells were retained if they a) contained ≥500 reads, b) contained ≤5% reads coming from mitochondrial genes, and c) were called a singlet by Demuxlet, leaving 23,676 cells for downstream analysis. Data were log-normalized, and then principal component analysis was run on variable features selected by the FindVariableFeatures function after scaling with regression of percentage mitochondrial and nReads per cell. UMAP and Louvain clustering were performed with Seurat defaults and resolution 0.3. Differential gene expression between subsets of PLZF^+^CD4^+^ T cells was performed using FindAllMarkers with Seurat’s MAST implementation (55). Genes were deemed significantly different if FDR < 0.05, if average fold-change > 1.2, and the gene was detected in > 5% of cells in either comparison group. Visualizations were created with dittoSeq (v1.6.0) (56). Gene set analyses were performed using Metascape (57) with visualizations created in R with ggplot2.

### CD4^+^ T cell stimulation assays.

Single-cell suspensions of primary CD4^+^ T cells isolated from the prenatal SI and MLNs were cultured in a 96-well U-bottom plate in complete RPMI (Gibco) supplemented with 10% FBS, 10 mM HEPES, 2 mM l-glutamine, and 1× nonessential amino acids (Gibco) and stimulated with plate-bound αCD3 mAb (clone HIT3a, BioLegend, catalog 300313) at 1 μg/mL and soluble αCD28 mAb (CD28.2, Invitrogen, catalog MA1-20792) at 2 μg/mL, and/or cells were activated with recombinant human IL-12 and IL-18 (R&D Systems), or IL-2 and IL-33, and/or IL-1β and IL-23 for 16–20 hours at 37°C in 4% O_2_, with Brefeldin A (eBioscience) added for the last 4 hours. All cytokines were used at 50 ng/mL and from PeproTech unless otherwise specified. After stimulation, cells were stained for intracellular cytokine production as described above. For allogeneic stimulation of prenatal CD4^+^ T cells, allogeneic CD14^+^ APCs were isolated from unrelated adult PBMCs with the CD14 Positive Selection Kit II (STEMCELL Technologies), plated at a density of 0.5 million CD14^+^ cells/well in a 96-well plate, and allowed to adhere. Nonadherent cells were removed after 30 minutes, and adherent cells were incubated with αMHC DP-DQ-DR mAb (BioLegend, clone Tü39) or isotype control (IgG2a, clone MOPC-173, BioLegend) at 10 μg/mL for 30 minutes at 37°C. Primary CD4^+^ T cells isolated from the prenatal SI were stained with CTV (Invitrogen) to differentiate these from residual adult T cells within CD14^+^ APCs and cocultured at a 4:1 (T cell/APC) ratio for 16 hours, with Brefeldin A added in the last 4 hours. Cells were stained for intracellular CD154 and cytokine production as described above.

### Naive CD4^+^ T cell expansion, maturation, and differentiation assays.

Primary naive CD4^+^ T cells obtained from mature CD4 SP thymocytes as described above were cultured with IL-7 (5 ng/mL), IL-2 (10 ng/mL), or IL-15 (10 ng/mL) for 7 days with media changes every 2–3 days. Cells were kept at 37°C and 4% O_2_ for the duration of the stimulation. In some cases, IL-7 doses were titrated as indicated, and/or TGF-β (10 ng/mL) was added for the duration of culture. TCR stimulation was provided by plate-bound αCD3 (clone HIT3a, BioLegend, catalog 300313) at 1 μg/mL unless otherwise specified and soluble αCD28 (clone CD28.2, BD, catalog 555725) at 2 μg/mL. For naive T cell maturation and differentiation assays, cells were washed and moved to a new 96-well plate at day 5–6, rested for 24 hours in the absence of cytokines, and matured further with IL-7 (5 ng/mL) and/or IL-2 (10 ng/mL) in the presence or absence of TCR stimulation as described above or under Th-skewing conditions for an additional 7 days. Cells were differentiated in the presence of TCR stimulation and IL-2 (10 ng/mL) for Th0, with the addition of IL-12 (2.5 ng/mL) for Th1, and the addition of IL-1b (10 ng/mL) and IL-23 (10 ng/mL) for Th17, in the presence or absence of IL-7 (5 ng/mL). For Th17 differentiation, cells were cultured in IMDM + 10% FBS with 1× nonessential amino acids, 2 mM l-glutamine, 10 mM HEPES, and penicillin/streptomycin. At day 14 of culture, cells were washed, replated, and rested for 24 hours as above, then restimulated with plate-bound αCD3 (1 μg/mL) and soluble αCD28 (2 μg/mL) or recombinant human IL-12 and recombinant human IL-18 (50 ng/mL) for 24 hours. Where indicated, cells were restimulated with PMA and ionomycin for 4 hours. Brefeldin A was added for the last 4 hours of each stimulation.

### Chemical inhibition.

Primary naive CD4^+^ T cells were labeled with CTV to track cell proliferation and cultured with IL-7 (10 ng/mL) in the presence or absence of chemical inhibitors at 37°C in 4% O_2_ and 5% CO_2_. Chemical inhibitors were used at the indicated concentrations and purchased from Selleck Chemicals unless otherwise indicated: 5 μM of SH-4-54 (catalog S7337), 500 nM PD0325901 (StemRD, catalog PD-010), 1 μM pictilisib/GDC-0941 (catalog S1065), 5 μM STAT3-IN-1 (catalog S0818), 50 μM JANEX-1 (catalog S5903), 10 μM solcitinib (catalog S5917), and 5 μM SIS3 (MilliporeSigma, catalog S0447-5MG). DMSO vehicle control was used at 1 μg/mL and 10 μg/mL. Expansion indices were defined as the fold-change in expansion of the overall culture and calculated in FlowJo. Expansion index is equivalent to the final count of proliferated cells divided by the starting cell count.

### Protein extraction and cytokine level quantification from solid tissue.

Fragments of tissue (10 mg) were taken from the SI lamina propria and MLNs and placed in chilled lysis buffer (1 μL/mL protease inhibitor, 3 μL/mL 500 mM PMSF in RIPA buffer). Tissue was disrupted by mixing 20 times with a 1 mL pipette tip cut to a 2 mm opening and then vortexed for 15 seconds. Tubes were shaken at 4°C for 20 minutes at 300 rpm, and then the entire process was repeated. Tissue was centrifuged at 400*g* for 10 minutes at 4°C and supernatant was collected. Tissue IL-7 and IL-15 concentrations were measured via cytokine bead array using the Human Hematopoietic Stem Cell Panel Kit (LEGENDplex). IL-12p70, IL-18, IL-1b, and IL-23 concentrations were measured using the Human Inflammation Panel 1 Kit (LEGENDplex).

### IL-7 ELISA.

Single-cell suspensions of SI IECs and SI lamina propria were obtained as described above, and live, lamina propria CD45^+^ and CD45^–^ cell fractions were sort-purified. Whole protein content was extracted from each of these populations as described above. Samples were kept on ice at all times. Whole protein concentrations were measured using the Pierce BCA Protein Assay kit (Thermo Fisher Scientific, catalog 23227) and stored at –80°C until further analysis. Samples were thawed on ice, normalized in 1× PBS, and subsequently analyzed for the presence of human IL-7 using the precoated IL-7 human ELISA kit (Thermo Fisher Scientific, catalog EHIL7) according to the manufacturer’s instructions.

### Immunofluorescence.

Immunofluorescence staining was performed on prefixed, whole SI sections, embedded in coils in OCT of prenatal samples (22-week gestational age). Tissues were sectioned to 10 μM using Leica cryostat CM 1950 set to –20°C. Tissue sections were later fixed in acetone for 5 minutes at –20°C, rehydrated in PBS for 10 minutes, rinsed with 0.05% TBS-Tween, and then permeabilized in 0.1% PBS–Triton X-100 for 15 minutes, all at room temperature in a humid container. Slides were then blocked for 1 hour at room temperature in a humid container in blocking buffer (10% calf serum, 1.5% BSA, 130 mM glycine, 1× Triton X-100 in PBS). Primary anti–IL-7 (IL-7 rabbit anti-human, Thermo Fisher Scientific: catalog PA519844) was used at 1:50 in blocking buffer and incubated overnight at 4°C in humid container, which was not included for control slides. Slides were washed 3 times for 30 minutes in PBS. Secondary anti-rabbit donkey in C555 antibody (Thermo Fisher Scientific, catalog 20038-1) was used at 1:300 in 0.05% PBS/Tween 20 for 2 hours at room temperature. Slides were washed 3 times for 30 minutes in blocking buffer. Slides were blocked with new blocking buffer (supplemented with 0.1% fish skin gelatin, 5% rat serum, and 1% donkey serum) overnight at 4°C in humid container. Preconjugated anti-CD4-APC (Becton Dickinson, mouse, clone RPA-T4, catalog 561841) was then applied for 4 hours at room temperature in supplemented blocking buffer at 1:200. Slides were washed 3 times for 30 minutes in PBS. Slides were then mounted with Fluoromount G + DAPI (Thermo Fisher Scientific, catalog 00-4959-52) and coverslipped. Slides were imaged immediately with ECHO microscope, using channels DAPI, Cy5, and Texas red at various exposures.

### Statistics.

Data were analyzed using Wilcoxon’s test for paired nonparametric data and 1-way ANOVA with post hoc Tukey’s honestly significant differences test for comparison of 3 or more groups. A *P* value less than 0.05 was considered significant. Box plot upper and lower hinges represent the first and third quartiles, the center line indicates the median, and the whiskers extend from the hinge to the highest and lowest value no further than 1.5× interquartile range from the hinge unless otherwise stated.

### Study approval.

PBMCs were isolated from Trima residues from Trima Apheresis collection kits and were obtained from healthy donors after receipt of written informed consent at the Blood Centers of the Pacific. Human prenatal tissues (16 to 22 weeks gestational age) were obtained from terminations of pregnancy after maternal written informed consent with approval from and under the guidelines of the UCSF Research Protection program. Samples were excluded in the cases of known maternal infection, intrauterine prenatal demise, and/or known or suspected chromosomal abnormality.

### Data availability.

All raw RNA-Seq data analyzed in this study have been deposited on NCBI GEO (GSE213522). Values for all data points found in graphs are in the Supporting Data Values file.

## Author contributions

VL and JH designed research studies. VL, SP, SM, MM, and JH conducted experiments. VL, SP, SM, MM, and JH acquired data. VL, SP, DGB, SM, MM, TDB, GKF, and JH analyzed data. VL and JH wrote the manuscript.

## Supplementary Material

Supplemental data

Supporting data values

## Version 1 10/19/2023

In-Press Preview

## Version 2 11/22/2023

Electronic publication
