# Supplementary material for: Homeostatic cytokines reciprocally modulate the emergence of prenatal effector PLZF+CD4+ T cells in humans
Source: JCI Insight. 2023 Nov 22;8(22):e164672. doi: 10.1172/jci.insight.164672 (PMC10721317; doi:10.1172/jci.insight.164672)
Supplement: Supplemental data [file jciinsight-8-164672-s194.pdf]

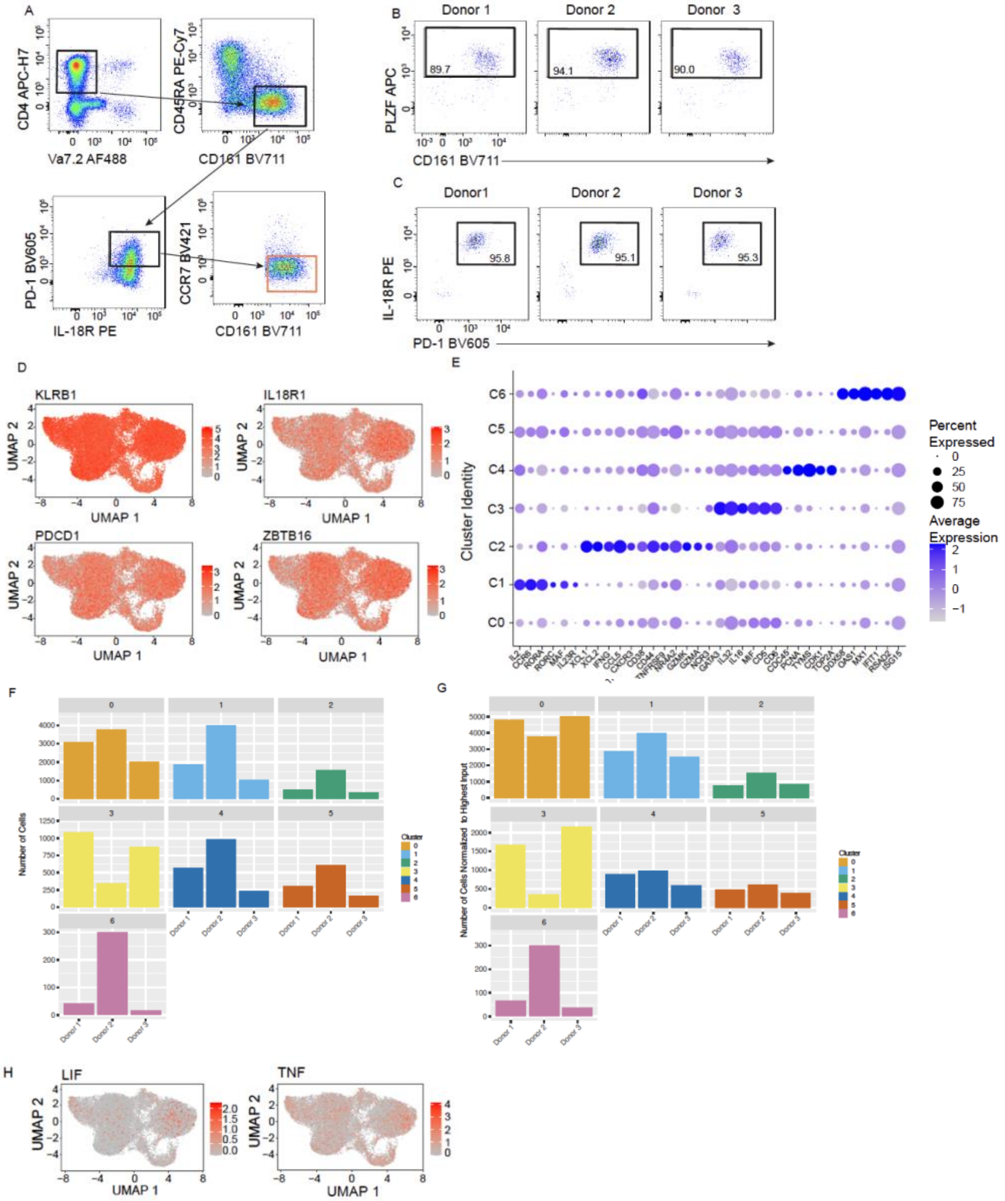

Supplemental Figure 1. Characteristics of sorted prenatal PLZF$^+$ CD4$^+$ T cells for scRNA-sequencing and representation of cluster compositions. (A) Sorting strategy for the identification and isolation of live, memory PLZF$^+$ CD4$^+$ T cells from the human prenatal SI. (B) Post-sort purity after intra-nuclear staining for PLZF and (C) expression of IL-18R and PD-1 for each of the donors submitted for scRNAseq. (D) UMAP visualization indicating ubiquitous expression of *KLRB1* (CD161), *IL18R1* (IL18R), *PDCD1* (PD-1) and *ZBTB16* (PLZF) after scRNAseq confirming identity of sort-purified PLZF$^+$ CD4$^+$ T cells. (E) Dot plot of selected DEG within identified clusters of PLZF$^+$ CD4$^+$ T cells. (F-G) Absolute (F) and normalized (G) cell number contribution per donor for each transcriptionally distinct PLZF$^+$ CD4$^+$ T cell cluster. When cell numbers are normalized to the highest input donor, individual contribution is equalized in 4 of the 6 clusters, indicating that inter-individual variation can partly be explained by differences in the number of cells contributed by each donor. (H) UMAP visualization demonstrating dispersed *LIF* and *TNF* expression across SI PLZF$^+$CD4$^+$ T cell clusters.

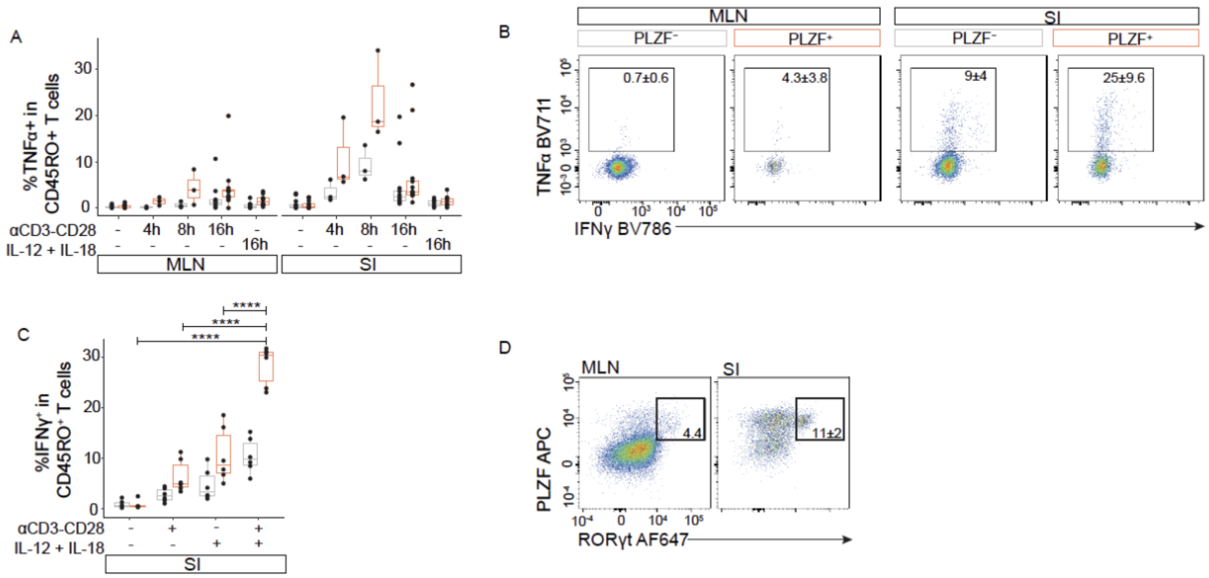

Figure S2: Prenatal PLZF$^+$ CD4$^+$ T cells are a functionally heterogeneous population. (A) Quantification of TNFα^+ cell frequencies after indicated stimulation conditions and durations among PLZF$^-$ (grey) and PLZF$^+$(orange) CD45RO$^+$ CD4$^+$ T cells isolated from cryopreserved fragments of prenatal SI and MLN. (B) Representative flow plots of peak intracellular TNFα^+ production after 8 hours of stimulation with anti-CD3/CD28. (C) Quantification of IFNγ^+ cell frequencies after indicated stimulation conditions among PLZF$^-$ (grey) and PLZF$^+$(orange) CD45RO$^+$ CD4$^+$ T cells isolated from cryopreserved fragments of prenatal SI indicating a synergistic effect of cytokine and TCR stimulation on IFNγ production. (D) Representative flow plots of RORγt expression among CD4$^+$ T cells isolated from the indicated prenatal tissues (n=3). Circles represent individual donors. Paired ANOVA with Tukey multiple comparison test (A,C). *$p < 0.05$, **$p < 0.01$, ***$p<0.001$ ****$p < 0.0001$,

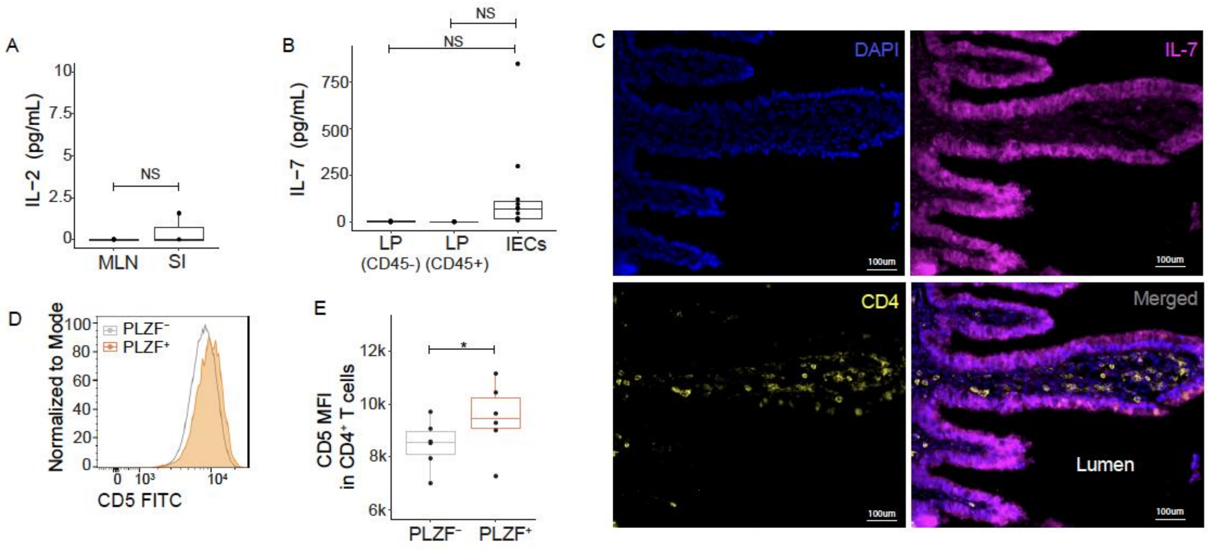

Figure S3: (A) Quantification of whole tissue concentration of IL-2 in the prenatal MLN and SI measured by cytokine bead array. (B) Concentrations of IL-7 protein calculated by ELISA within different compartments of the prenatal SI indicating detectable levels within EDTA-isolated intestinal epithelial cells (IECs; n=11) with minimal IL-7 production in either sort-purified $CD45^+$ (n=3) or $CD45^-$ (n=3) cell fractions in the lamina propria. (C) Representative image (n=3) at 20X magnification of prenatal small intestine showing immunostaining with antibodies to IL-7 (magenta), CD4 (yellow) and DAPI (blue) indicating diffuse IL-7 expression in the epithelial compartment of the prenatal small intestine with CD4+ cells localizing to the subepithelial space of the lamina propria (n=3). (D) Representative histogram and (E) MFI of CD5 expression among indicated populations of naïve $CD4^+$ T cells isolated from the prenatal thymus. (A,C,E) Circles represent individual donors. Wilcoxon Signed Rank Test (A,E) and paired ANOVA with Tukey multiple comparison test (B). *p < 0.05, **p < 0.01, ***p<0.001 ****p < 0.0001.

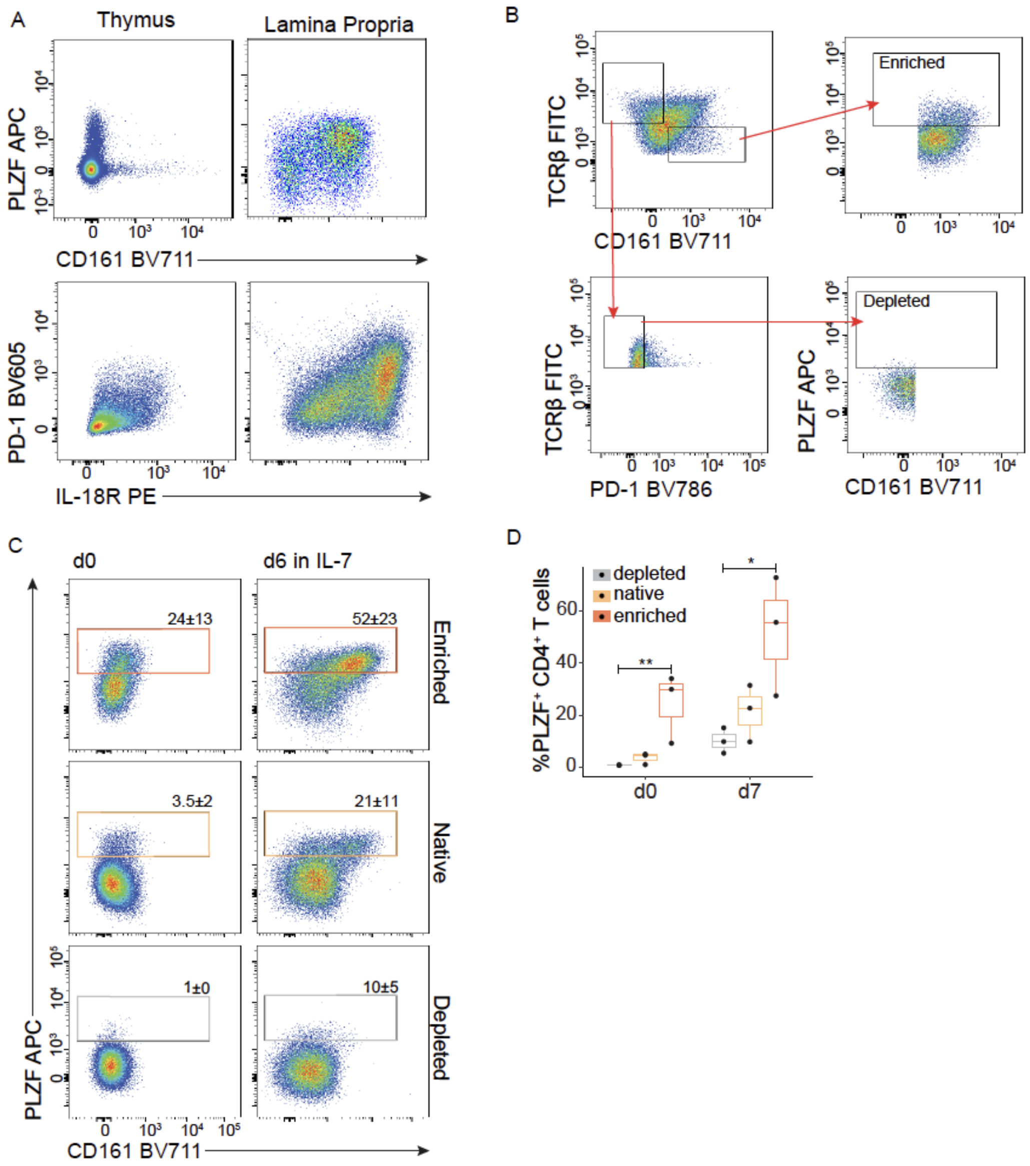

Figure S4: New sorting strategy for enrichment and depletion of naïve PLZF$^+$ cells from CD4$^+$ naïve T cells in the thymus. (A) Representative flow plots indicating limited expression of CD161 (top) and PD-1 and IL18R (bottom) among naïve CD4$^+$ T cells in the thymus compared to memory CD4$^+$ T cells in the SI drive the need for an alternative approach for the separation of PLZF$^+$ from PLZF$^-$ subsets of naïve CD4$^+$ T cells in the thymus. (B) Sorting strategy to enrich (TCR$\alpha\beta^{lo}$ CD161$^+$) and deplete (TCR$\alpha\beta^{hi}$ CD161$^-$ PD1$^-$) for naïve PLZF$^+$ CD4$^+$ T cells from mature CD1a$^-$ TCRab$^+$ CD4 single positive thymocytes. (C) Representative post-sort flow plots and (D) quantification of naïve PLZF$^+$ CD4$^+$ T cells before initiation of culture (d0) and after 6 days of culture with 10ng/mL IL-7. Starting “Enriched” and “Depleted” cell populations were sorted using the strategy in S4B. “Native” population refers to all mature CD1a$^-$ TCR$\alpha\beta^+$ CD4 single positive thymocytes. (D) Circles represent individual donors. Paired ANOVA with Tukey multiple comparison test (D). *$p < 0.05$, **$p < 0.01$, ***$p<0.001$ ****$p < 0.0001$

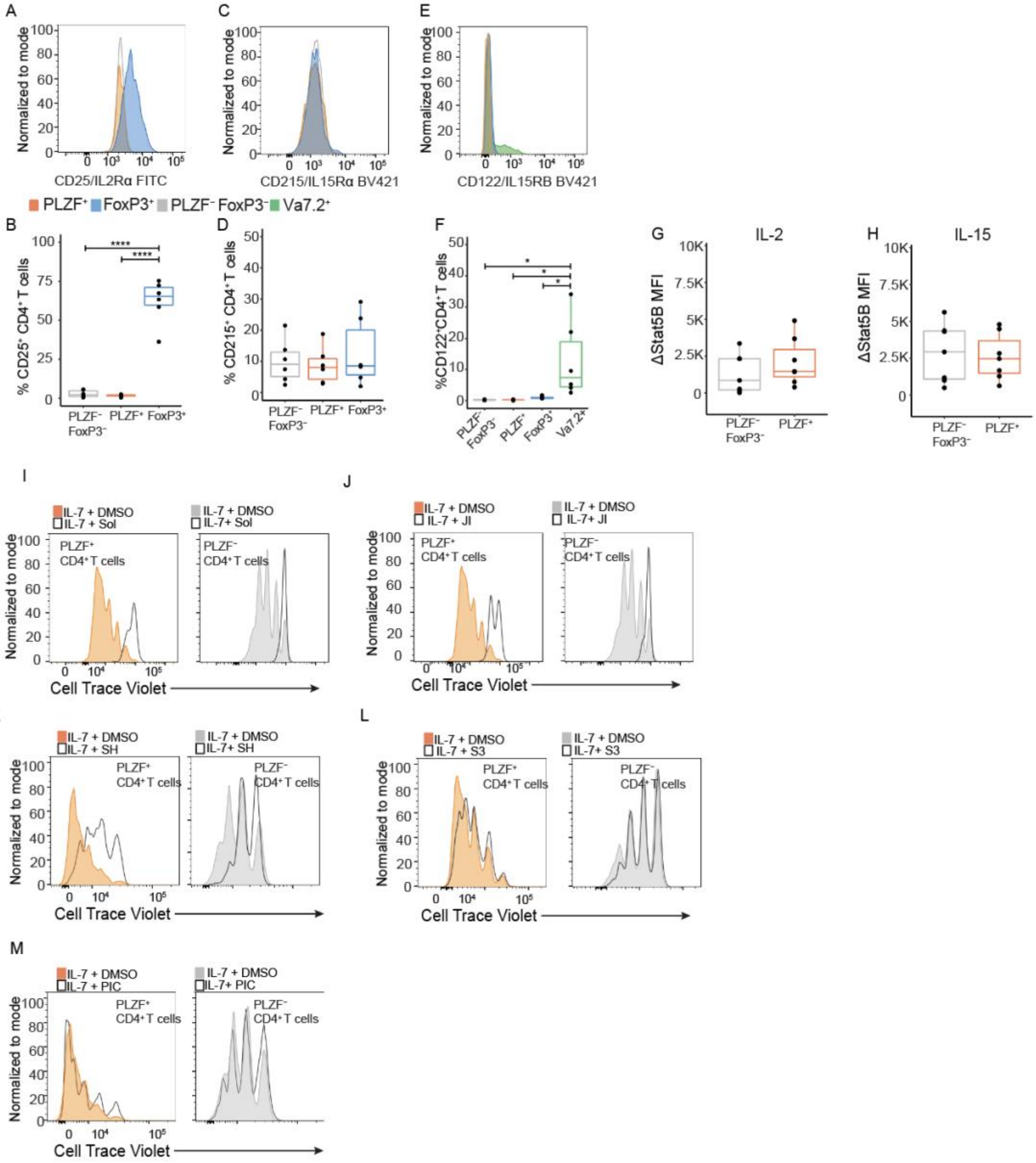

Figure S5: Characteristics of cytokine signaling in prenatal naïve CD4⁺ T cell subsets. (A, C, E) Representative histograms and (B, D, F) frequencies of CD25, CD215, and CD122 expression within indicated populations of naïve CD4⁺ T cells in the thymus. (F) Comparison is made to IL15RB-expressing semi-invariant innate-like T cells (CD3⁺Vα7.2⁺, indicated as Vα7.2⁺) in the thymus. (G, H) Normalized MFI of pSTAT5B phosphorylation in prenatal PLZF⁺ or PLZF⁻ CD4 naïve T cells after stimulation for 30 minutes with (G) IL-2 or (H) IL-15. (I,J,K,L,M) Effect of small molecule signaling inhibitors on the proliferation of (left) PLZF⁺ or (right) PLZF⁻ subsets of CD4 naïve T cells. Representative histograms of cell trace violet (CTV)-dilution of indicated naïve CD4 T cell populations after 6-day culture with IL-7 in the presence of the indicated chemical inhibitors (open histograms) compared to DMSO controls (tinted histograms). Inhibitors used: (I) Sol (Solcitinib, JAK1), (J) J1 (JANEX-1, JAK3), (K) SH (SH-4-54, STAT3 and STAT5), (L) S3 (STAT3-IN-1, STAT3), (M) PIC (Pictilisib, PI3Kα/δ). Circles represent individual donors. Paired ANOVA with Tukey's multiple comparison test (B, D, F) and Wilcoxon Signed Rank Test (G, H) *p < 0.05, **p < 0.01, ***p<0.001 ****p < 0.0001.

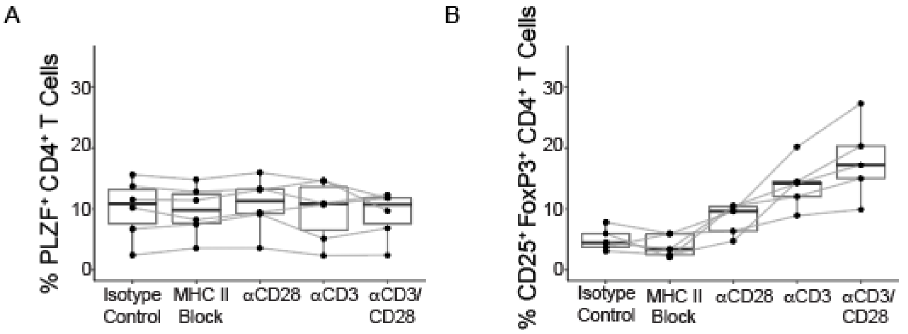

Figure S6: TCR signaling does not interfere with the IL-7-driven expansion of naïve PLZF$^+$CD4$^+$ T cells.
Proportion of (A) PLZF$^+$ CD4$^+$ T cells and (B) CD25$^+$FoxP3$^+$ CD4$^+$ T cells generated after 6 days of naïve CD4$^+$ T cell culture with (A) IL-7 or (B) IL-2 in the presence of indicated additives shows PLZF$^+$ CD4$^+$ T cells accumulate to similar proportions in the presence or absence of TCR signaling. Conversely, the combination of anti-CD3/anti-CD28 stimulation enhances the generation of CD25$^+$FoxP3$^+$ CD4$^+$ T cells. Circles represent individual donors.

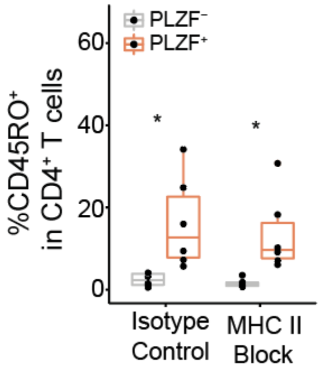

Figure S7: Emergence of CD45RO$^+$ CD4$^+$ T cells in response to IL-7 is unaffected by inhibition of low-level TCR signaling from T cell-T cell interactions. The frequencies of memory (CD45RO$^+$) T cells that accumulate among indicated CD4$^+$ T cell subsets after 6 days of culture of naïve CD4 T cells with IL-7 in the presence of MHC-II blocking antibody are similar to those treated with isotype control (n=6). Circles represent individual donors. Paired ANOVA with Tukey's multiple comparison test. *p < 0.05, **p < 0.01, ***p<0.001 ****p < 0.0001.

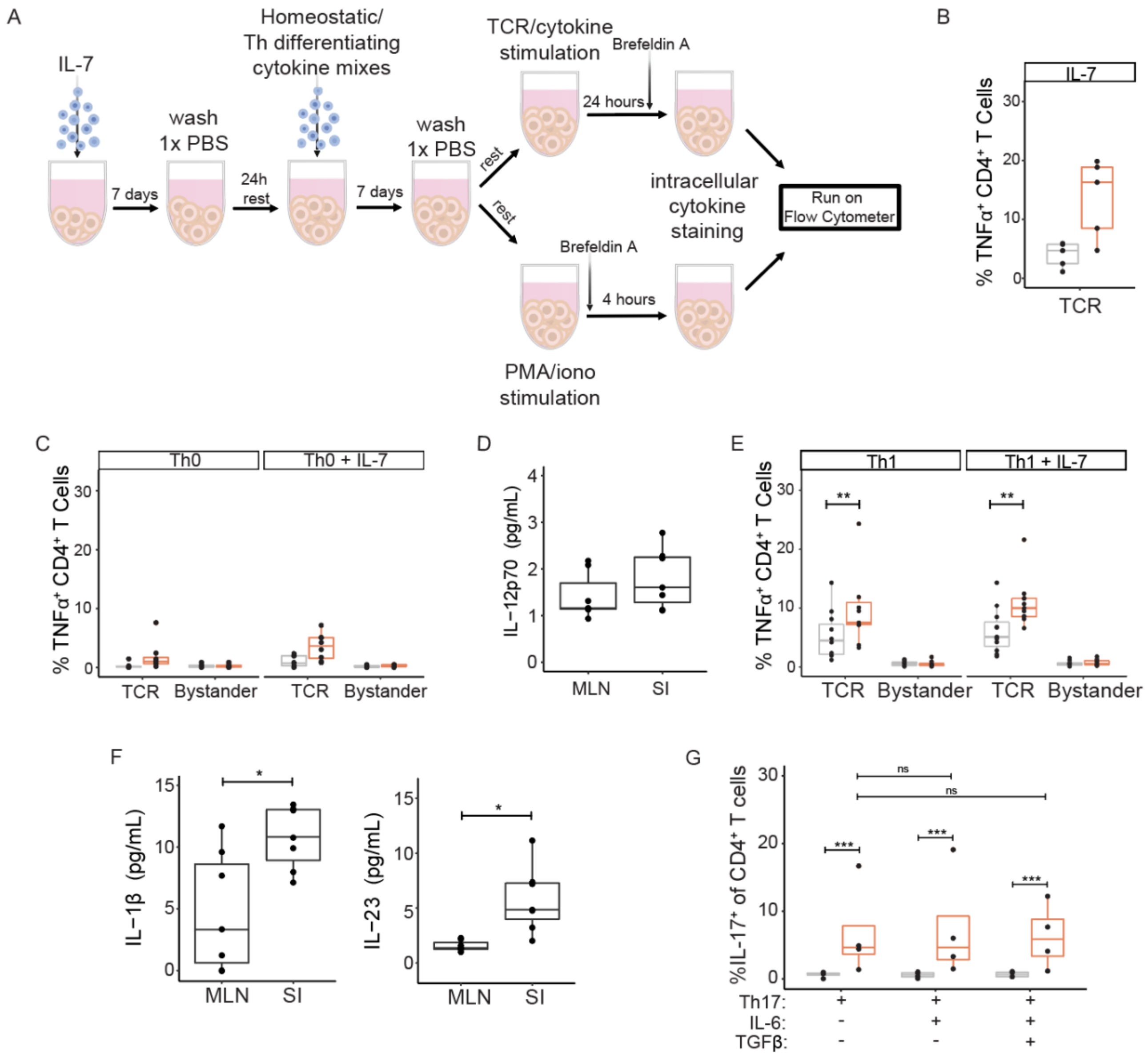

Figure S8: Homeostatic cytokines specifically contribute to the effector maturation of prenatal PLZF$^+$ CD4$^+$ T cells.

(A) Schema of the two-step in vitro culture system in which naïve CD4$^+$ T cells were first expanded in the presence of IL-7 alone, followed by 7 days of maturation in the indicated conditions: IL-7 alone, IL-7 + TCR (in the presence of αCD3/CD28), Th0 (TCR + IL-2); Th1 (TCR + IL-12), Th17 (TCR + IL-23 + IL-1β) used in Figure 7A-F. (B, C, E) Proportions of TNFα$^+$ and (G) IL-17$^+$ cells among CD4$^+$ T cells matured in the indicated conditions following 24-hour re-stimulation with αCD3/CD28 (TCR) or IL-12/IL-18 (Bystander). (D, F) Quantification of whole tissue concentrations of (D) IL-12p70, (F) IL-1β, and IL-23 levels in the prenatal MLN and SI measured by cytokine bead array. (G) Proportions of IL-17$^+$ cells among CD4$^+$ T cells matured in the indicated conditions following 4-hour stimulation with PMA/Ionomycin. Brefeldin A was added in the final 4 hours of all re-stimulations. Circles represent individual donors. Wilcoxon Signed Rank Test (B-G) *$p < 0.05$, **$p < 0.01$, ***$p<0.001$ ****$p < 0.0001$.
